# Anti-Inflammatory Dietary Supplements in Pregnancy: A Scoping Review

**DOI:** 10.3390/nu18213627

**Published:** 2026-10-31

**Authors:** Anamaria Apan, Cristina Pop, Irina Iaru, Oliviu Voștinaru, Răzvan Mircea Cherecheș, Maria Bonea, Cristina Mogoșan

**Affiliations:** 1 Department of Pharmacology, Physiology, Pathophysiology, Faculty of Pharmacy, “Iuliu Hațieganu” University of Medicine and Pharmacy, 400012 Cluj-Napoca, Romania; anamaria.apan@umfcluj.ro (A.A.); cristina.pop.farmacologie@gmail.com (C.P.); oliviu_vostinaru@yahoo.com (O.V.); cmogosan@umfcluj.ro (C.M.); 2 HIVE Health Innovation Center, Babeș-Bolyai University, 400095 Cluj-Napoca, Romania; razvan.chereches@ubbcluj.ro; 3 Department of Neurosciences—Psychiatry and Pediatric Psychiatry, Faculty of Medicine, “Iuliu Hațieganu” University of Medicine and Pharmacy, 400012 Cluj-Napoca, Romania; bonea.maria@umfcluj.ro

**Keywords:** maternal nutrition, nutraceuticals, vitamin D, omega-3 fatty acids, probiotics, gestational diabetes mellitus

## Abstract

Purpose: This scoping review aimed to map the available evidence on the safety and efficacy of anti-inflammatory dietary supplements used during pregnancy, assessing maternal and fetal outcomes. Methods: A systematic literature search was conducted in PubMed, Scopus, and Web of Science (January 2016–June 2026), following PRISMA-ScR guidelines. The protocol was prospectively registered on the Open Science Framework (10.17605/osf.io/4kmv9). Eligible studies included human clinical trials and observational studies evaluating anti-inflammatory dietary supplements in pregnant women. Results: Eighty unique studies (96 publications) from 28 countries were included, of which 63 (78.8%) were randomized clinical trials. Thirteen supplement categories were identified. Vitamin D (*n* = 23) and omega-3 fatty acids (*n* = 17) accounted for nearly half of all studies; probiotics (*n* = 11) formed the third-largest category. For these three supplements, most studies reported changes in inflammatory and glycemic markers, primarily in gestational diabetes mellitus and high-risk populations, but adverse events were collected in a minority of studies, so tolerability is documented rather than established. Evidence for zinc, magnesium, and alpha-lipoic acid was limited. Curcumin, chlorella, vitamin E, epigallocatechin-3-gallate (EGCG), resveratrol, and ginger were each supported by only one or two studies. Supplementation was initiated in the first trimester in 19 studies (23.8%), and neonatal safety outcomes were reported infrequently. Conclusions: Evidence on anti-inflammatory dietary supplements in pregnancy is concentrated in a few compounds, in a small number of countries, and in biochemical outcomes measured in selected populations. Tolerability was evaluated in only a small number of studies, which limits its use as an indicator of safety. The map highlights the scarcity of controlled studies with prespecified harm outcomes, of first-trimester initiation, and of infant follow-up beyond the first months of life.

## 1. Introduction

Pregnancy represents a complex biological and developmental stage in a woman’s life when the mother-to-be is paying extra attention to diet and supplementation to improve her own health and prevent negative impacts on her unborn child [1,2]. Nutritional status plays a crucial role during the developmental period, influencing lifelong disease risk. Because of that, it is considered a modifiable risk factor [3].

In some cases, the evidence regarding the need for and importance of supplementation during pregnancy is well established, because deficiencies in essential nutrients can lead to birth defects [1,2,4]. For example, folic acid reduces the risk of neural tube defects, iron supplementation prevents pregnancy-related anemia, and iodine supplementation supports healthy thyroid function in the fetus and prevents neurocognitive disorders. The guidelines for antenatal care provided by the World Health Organization (WHO), the American College of Obstetricians and Gynecologists (ACOG), and the National Institute for Health and Care Excellence (NICE) acknowledge the benefits of, and need for, supplementation before and during pregnancy [3,5,6,7]. Dietary supplement use is common during pregnancy, ranging from 77% to 92% of pregnant women, using primarily conventional supplements like prenatal vitamins, folic acid, and iron [8]. The use of non-conventional dietary supplements, notably herbal supplements and specialty tea, was reported by 13% of pregnant women [9].

Given the relatively high incidence of nutritional deficiencies, the low nutrient density of foods and the increased consumption of nutritionally poor processed foods, supplementation during pregnancy may be beneficial in some cases. However, adverse effects may occur: excessive vitamin A or carotenoid intake increases the risk of birth defects, papain increases miscarriage risk, phytoestrogens (isoflavones) from soy produce estrogen-like effects, and excessive vitamin E and C supplementation increases the risk of fetal loss and premature rupture of membranes [10,11].

Beyond WHO-recommended supplements for all pregnant women (iron, folates) and those it recommends only in context-specific settings (vitamin A—in areas where vitamin A deficiency is a severe public health problem or to prevent night blindness; calcium—in populations with low dietary calcium intake; and zinc—in the context of rigorous research), while vitamin D is not recommended for pregnant women outside research settings, more research is needed to determine if other micronutrients can improve maternal and fetal outcomes [5]. However, the global prenatal supplements market has grown into a profitable industry, with a projected compound annual growth rate of 8.4% from 2024 to 2034, driven by aging maternal populations, increased awareness of prenatal nutrition, and rising prevalence of nutritional deficiencies [12]. Despite conflicting public information on safety and efficacy, consumption continues to grow [2,13].

The legal framework that applies to dietary supplements is not very well defined in some countries, and a significant difference exists between the United States and the European Union. However, these two systems have one thing in common: neither requires pre-market studies to demonstrate the efficacy and safety of these products. In the United States, the Dietary Supplement Health and Education Act of 1994 (DSHEA) is responsible for the regulation of dietary supplements, and the Food and Drug Administration (FDA) does not have the authority to approve dietary supplements; it intervenes only after the dietary supplements reach the market and only if the product presents a risk to the population [14]. Within the European Union, food supplements are primarily regulated by Directive 2002/46/EC, which classifies them as dietary products intended to supplement the normal diet and includes a list of vitamins, minerals, and their sources. The European Food Safety Authority (EFSA) carries out scientific risk assessments of substances used in dietary supplements and assesses health claims in accordance with Regulation (EC) No 1924/2006. In addition to the listed vitamins and minerals, other substances commonly found in supplements, including amino acids, essential fatty acids, and herbal extracts, are not yet subject to regulation at the EU level and instead may be subject to national regulations or the Novel Food Regulation (EU 2015/2283) [15,16]. In Europe, food supplements that do not comply with food law or that present a risk are notified by the national food safety authorities of the member states through the Rapid Alert System for Food and Feed (RASFF). RASFF notifications are market-surveillance records exchanged between authorities, not reports of adverse events in consumers. Between 2016 and 2026, the RASFF received 2286 notifications regarding dietetic foods, food supplements, and fortified foods, of which 304 notifications concerned high content of vitamins/minerals, contamination with other substances, or unauthorized novel food ingredients. From 304 alerts, 51 notifications were regarding high content of vitamin D, vitamin A, zinc, iron, iodine, folic acid, curcumin, ginger and berberine in supplements that could be used by pregnant women [17].

No additional regulatory requirements apply to supplements explicitly marketed for pregnant women, despite their classification as a vulnerable population [18].

The category of dietary supplements for pregnant women is rapidly increasing, and supplements that claim to have anti-inflammatory effects deserve special attention, given the important role that inflammation plays in the physiology of pregnancy. Pregnancy is not associated with a decrease in the activation of the immune system; on the contrary, the immune system is one of the most important systems of defense for the mother and fetus against potential damaging factors; therefore, pregnancy is a pro-inflammatory and anti-inflammatory condition, depending on the gestational stage [19]. The first trimester of pregnancy is characterized by a pro-inflammatory state, necessary for embryo implantation; the second trimester is characterized by an anti-inflammatory state, supporting fetal growth; and the third trimester returns to a pro-inflammatory state, which prepares the organism for delivery [19,20]. Exacerbated inflammation during pregnancy is a major concern, with possible consequences for the continuation of pregnancy [21]. Elevated proinflammatory cytokines, including interleukin (IL)-6, IL-1β, and tumor necrosis factor (TNF)-α, were observed in mothers with preeclampsia and fetal growth restriction. Elevated glucose levels promote a pro-inflammatory maternal–fetal environment characterized by increased production of pro-inflammatory cytokines, which could lead to gestational diabetes mellitus; a maternal inflammatory state may also contribute to neurodevelopmental disorders in children [21,22].

This inflammatory vulnerability has provided a rational justification, sometimes supported by evidence, for the use of dietary supplements with anti-inflammatory properties in pregnancy. Omega-3 fatty acids, specifically docosahexaenoic acid (DHA) and eicosapentaenoic acid (EPA), modulate prostaglandin pathways, with evidence supporting reduced preterm birth risk and potential neurological benefits [23,24]. Vitamin D acts as an immunomodulator and has been shown to reduce the risk of preeclampsia and gestational diabetes in clinical trials, although the results are inconsistent [25]. N-acetylcysteine (NAC), a glutathione precursor, showed promising results in preclinical models by reducing fetal cytokine responses [26], while curcumin, which inhibits nuclear factor (NF)-κB signaling and decreases pro-inflammatory cytokines, showed protective effects against adverse pregnancy outcomes in animal models [27,28]. Due to their influence on the intestine-immune system axis, probiotics have been the subject of several studies evaluating their ability to modulate systemic inflammation during pregnancy [29]. Choline supplementation in pregnancy positively influenced fetal brain development by decreasing maternal C-reactive protein (CRP) [30].

The underlying mechanism of action of each of these supplements is reasonable, but a reasonable mechanism of action does not necessarily constitute clinical evidence. For most anti-inflammatory supplements used during pregnancy, the available data are limited, with studies covering a variety of different compounds, outcome measures, study protocols, and populations. Efficacy has been studied more often and more rigorously than safety, and the interaction between anti-inflammatory supplements and inflammatory processes in pregnancy remains poorly characterized. Moreover, regulatory requirements do not demand that these concerns be addressed before market approval. This scoping review aims to map the published clinical evidence on anti-inflammatory dietary supplements used during pregnancy, assessing outcomes for the mother and fetus, as well as tolerability and adverse-event data, where reported. This review seeks to highlight areas where evidence is robust enough to support clinical practice, those where data are emerging but incomplete, and those where data are lacking, thereby providing a foundation for future research.

## 2. Materials and Methods

This scoping review was conducted following PRISMA-ScR (Preferred Reporting Items for Systematic Reviews and Meta-Analyses extension for Scoping Reviews) guidelines [31,32]. The protocol was registered on the Open Science Framework (OSF; DOI 10.17605/osf.io/4kmv9), and the literature search was conducted in accordance with the registered protocol. The search was limited to human studies published in English and was performed on 5 June 2026 across PubMed, Scopus, and Web of Science (1 January 2016—1 June 2026), using combinations of terms for (1) pregnancy, (2) anti-inflammatory dietary supplement, (3) maternal/fetal outcomes, and (4) clinical study designs. The PubMed search was repeated on 14 October 2026 after correction of errors identified in the executed strategy. Additional sensitivity searches were performed in Scopus and Web of Science, and records not retrieved by the main searches were deduplicated against the screened library and screened using the same eligibility criteria. The sensitivity searches yielded 4 further eligible studies. The eligibility criteria for included studies, the full database-specific search strategies, record counts, and deduplication steps are provided in the Supplementary Material, Tables S1 and S2.

Data were extracted from the included studies using a predefined form in Microsoft Excel (Microsoft Corporation, Redmond, WA, USA) and visualized using Python (version 3.13.15) with the matplotlib library (version 3.10.0) in the Google Colaboratory environment (Google LLC, Mountain View, CA, USA). Extracted data included study details, country, study setting, sample size, participant characteristics, the dietary supplement administered and its characteristics, and maternal and fetal outcomes. When companion papers from the same trial reported different outcomes, they were included as linked publications of the same study and analyzed together. Publications were linked to a single study when they shared a trial registration number or, in its absence, the same setting, recruitment period, and sample size (Supplementary Material, Table S3).

## 3. Results

### 3.1. Search Results and Study Selection

A total of 12,973 results were retrieved after a systematic search of the literature across three databases: 2280 from PubMed, 5154 from Scopus, and 5539 from Web of Science. Prior to screening, 6811 duplicate records and 21 retracted publications were identified and removed, resulting in 6141 studies that were subsequently organized and screened using Rayyan (Rayyan Systems Inc., Cambridge, MA, USA; https://www.rayyan.ai) [33] and Microsoft Excel: 3889 from the first Scopus and Web of Science searches and the corrected PubMed search, and 2252 added by the sensitivity searches in Scopus and Web of Science. Records that failed the eligibility criteria for population, intervention, outcome, or study design were excluded (*n* = 5977).

**Figure 1:**
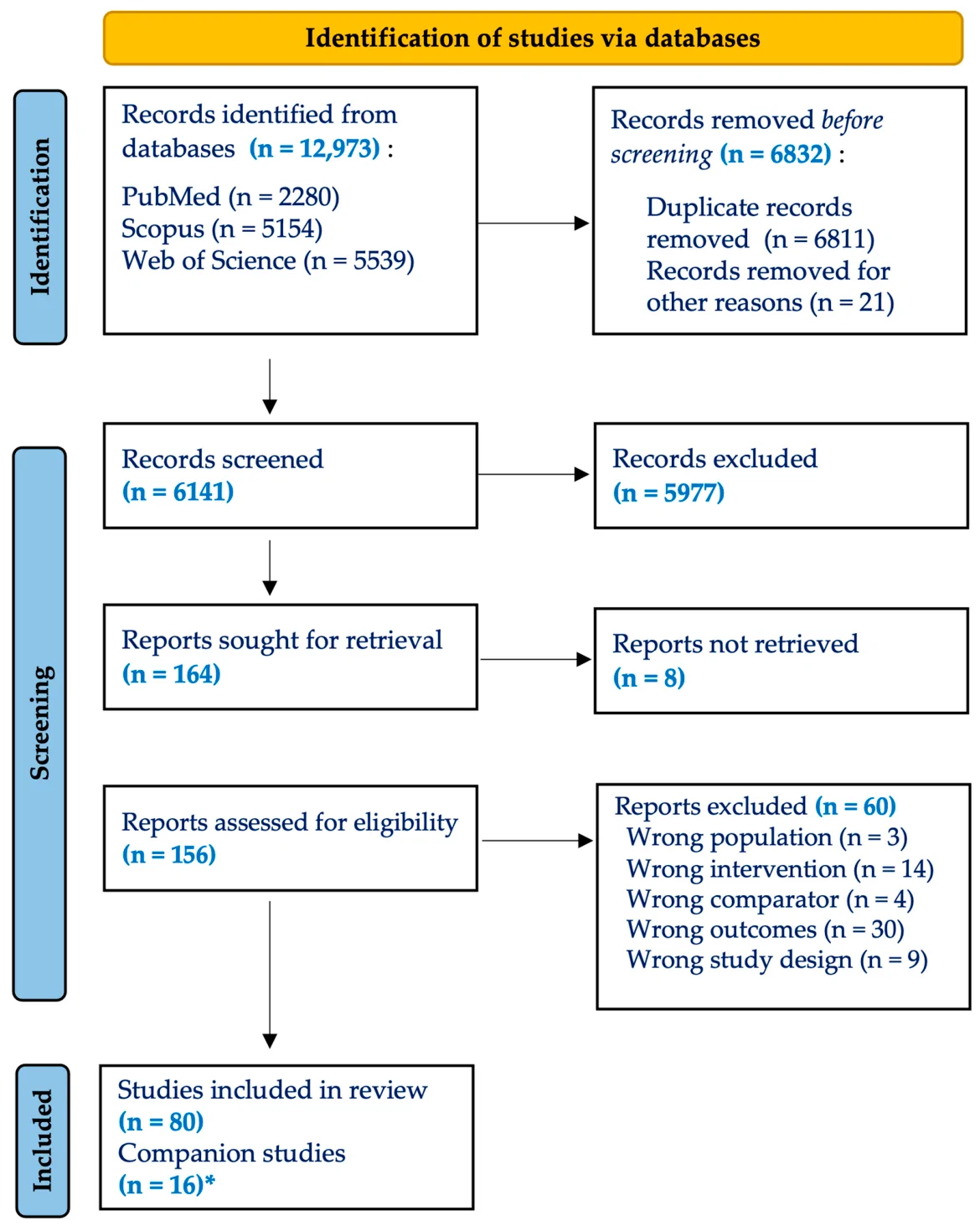
PRISMA Flow Diagram (* the 16 companion publications belong to 12 study families).

One hundred and sixty-four reports were sought for retrieval; eight could not be retrieved, and 156 full-text reports were assessed for eligibility. Sixty reports were excluded, and 80 unique studies, comprising 96 publications, met the inclusion criteria and were incorporated into the review (Supplementary Material, Table S3). Sixteen publications were companion papers reporting additional outcomes from the same trial population, belonging to 12 study families. Figure 1 depicts the complete study selection process.

Throughout this section, all counts refer to unique studies (*n* = 80), unless otherwise specified, and are based on the primary publication of each study. Sixteen publications were companion papers reporting additional outcomes from 12 of these studies, giving a total of 96 publications.

### 3.2. Characteristics of Included Studies

#### 3.2.1. Study Details

Studies were from 28 countries, which were unevenly represented. Most of them were from West Asia (*n* = 32, 40.0%) [34,35,36,37,38,39,40,41,42,43,44,45,46,47,48,49,50,51,52,53,54,55,56,57,58,59,60,61,62,63,64,65,66,67,68,69,70,71], dominated by Iran (*n* = 28) [36,37,38,39,40,43,44,45,46,47,48,49,50,51,52,53,54,55,56,57,58,59,60,61,62,63,64,65,66,67,68,69,70,71], followed by East and Southeast Asia (*n* = 18, 22.5%) [72,73,74,75,76,77,78,79,80,81,82,83,84,85,86,87,88,89], especially China (*n* = 11, 13.8%) [73,75,78,79,81,84,85,86,87,88,89], Europe accounted for 12 studies (15.0%) [90,91,92,93,94,95,96,97,98,99,100,101,102,103,104,105,106], of which three were conducted in Italy [96,101,106] and one was a European multi-center study [107,108]. Ten studies (12.5%) were conducted in North and South America [109,110,111,112,113,114,115,116,117,118,119,120], six of them in the United States of America [109,114,115,116,117,118,119]. Australia and New Zealand accounts for three studies (3.8%) [121,122,123,124,125] and South Asia for four studies (5%) [126,127,128,129] (Supplementary Material, Table S3).

Most of the included studies were randomized clinical trials (RCTs) (*n* = 63, 78.8%), including one randomized pilot trial. Three studies were secondary analyses of RCTs (3.8%), seven were retrospective-cohort studies (8.8%), and five (6.3%) were non-randomized controlled intervention studies. One (1.3%) was an uncontrolled prospective single-arm feasibility study [100], and one (1.3%) was a case report [105]. The last two have no comparator and are reported throughout as descriptive safety reports, separately from the 78 comparative studies.

#### 3.2.2. Participants Characteristics

Studies included pregnant women with or without comorbidities. The most common was gestational diabetes mellitus (GDM): 20 studies (25%) [36,37,38,39,40,45,46,47,48,49,50,51,52,53,54,55,61,70,87,88,89,106,128] enrolled women diagnosed with GDM, confirmed by the oral glucose tolerance test (OGTT) at 24–28 weeks, on insulin therapy or no medication. One study included women with impaired glucose tolerance (IGT) [63,64], five studies included mixed populations, and 43 studies (53.8%) included women without GDM.

Preeclampsia or gestational hypertension (*n* = 9, 11.8%) and overweight/obesity (*n* = 10, 13.2%; BMI ≥ 25 or ≥30 kg/m^2^) were the next most frequent comorbidities [35,41,44,60,65,72,73,74,75,92,93,95,102,111,117,126].

Six studies included pregnant women at risk for preterm birth (7.9%), and other specific comorbidities were vitamin D deficiency, depression or anxiety during pregnancy, gastrointestinal discomfort, recurrent miscarriage and risk of intrauterine growth restriction (IUGR). Nineteen studies (25%) included pregnant women without major comorbidities, recruited from routine prenatal care centers.

#### 3.2.3. Intervention Characteristics

Most studies evaluated a single supplement (*n* = 70, 87.5%), including 12 individual dietary supplements with anti-inflammatory activity: omega-3 fatty acids (algal oil, fish oil, EPA/DHA) [34,43,44,50,61,78,79,80,84,109,111,113,114,115,116,118,120,123,124,127], vitamin D [35,41,42,53,57,58,59,65,68,69,70,77,85,86,87,88,90,93,94,95,102,103,105,107,108,119,129], vitamin E [72], zinc [56,63,64,66,71,75,128], magnesium [92,110,112], curcumin [74], ginger [40,100], epigallocatechin-3-gallate (EGCG) [81,89], probiotics [39,45,46,47,51,67,76,91,96,97,121,122,125], alpha-lipoic acid [36,37,38,55], chlorella [83], and resveratrol (as adjunctive therapy in two studies) [73,101]. The remaining 10 studies (12.5%, 12 publications) focused on combinations of dietary supplements (prebiotic + probiotic, omega-3 + vitamin E, omega-3 + vitamin D, omega-3 + probiotic, omega-3 + anthocyanins + alpha-cyclodextrins, magnesium + vitamin E, vitamin E + vitamin C) [48,49,52,53,54,60,62,98,99,104,106,117,126].

Vitamin D and omega-3 fatty acids accounted for half of the studies included in the present analysis (*n* = 40, 50%). Supplementation was most often initiated in the second trimester (T2) (*n* = 42, 52.5%), and less frequently in the first (T1) (*n* = 19, 23.8%) or third trimester (T3) (*n* = 9, 11.3%). In 10 studies (12.5%), the initiation window spanned more than one trimester or was not specified (Table 1). When these mixed labels are counted, some pregnant women started supplementation in the first trimester in 25 studies (31.3%). The trimester refers to initiation, not to the period of exposure, which in most studies continued to delivery. The duration ranged from 16 to 37 weeks, depending on gestational age at enrollment.

#### 3.2.4. Comparator

Placebo was the comparator in 51 studies (63.8%), passive control/no treatment in 16 studies (20.0%), and an active comparator consisting of a different dose of vitamin D or another form of dietary supplement in 10 studies (12.5%). One trial had both an active and a placebo arm, and in two descriptive safety reports no comparator was used.

#### 3.2.5. Maternal and Fetal/Neonatal Outcomes

Seventy-nine of the 80 studies assessed maternal outcomes, and 59 assessed fetal/neonatal outcomes. Maternal outcomes included biochemical outcomes—inflammatory markers (*n* = 25; IL, TNF-α, CRP), lipid profile (*n* = 21; HDL-C, LDL-C, total cholesterol, triglycerides) and glucose metabolism parameters (*n* = 29; fasting blood glucose, insulin levels, HbA1c, HOMA-IR)—and clinical outcomes, namely gastrointestinal symptoms (*n* = 25), GDM (*n* = 19), preeclampsia/gestational hypertension (*n* = 41), gestational weight gain (*n* = 13), preterm birth (*n* = 40, including women with preterm birth risk factors), mode of delivery (cesarean section, *n* = 31), and other maternal outcomes including oxidative stress and depressive symptoms. Adverse events were collected in 26 studies (32.5%): serious adverse events were prespecified and monitored in six (four omega-3 trials, one probiotic pilot trial and one vitamin D dose-comparison trial), and adverse events or tolerability were actively collected in 20 others, one of which monitored symptoms but did not report the results. Fifty-four studies (67.5%) did not report whether adverse events were collected.

Fetal/neonatal outcomes included birth weight (*n* = 50), Apgar score (*n* = 24), small for gestational age (SGA)/large for gestational age (LGA) or macrosomia (*n* = 20), congenital anomalies (*n* = 8), and other outcomes (head circumference, birth length, neonatal intensive care unit (NICU) admission, perinatal mortality, neonatal hypoglycemia, hyperbilirubinemia). Two publications reported infant outcomes beyond the neonatal period, at 4–8 weeks [68] and at approximatively 3 months of age [114].

### 3.3. Findings by Supplement Category

Unless otherwise specified, all numbers refer to the 80 unique studies. Each study’s results are presented as the authors reported them, without any evaluation. The term “significant” is used according to the statistical tests conducted by the original researchers. Biochemical outcomes are described as changes in biomarker concentrations. Tolerability is assessed based on gastrointestinal symptoms and adverse events, with the number of studies that collected such data specified in each case.

#### 3.3.1. Vitamins

Vitamin D (*n* = 23)

Vitamin D was the most extensively studied supplement across populations, including healthy pregnant women, those with GDM, vitamin D deficiency or insufficiency, or at risk for preeclampsia. Doses ranged from 200 IU/day to 4000 IU/day, with intermittent high-dose regimens (50,000 IU every two weeks), administered orally. Three trials compared two or more doses [35,58,85,88,129], and one compared supplementation with sun exposure and with routine care [82].

Maternal biochemical outcomes. Inflammatory marker responses were heterogeneous. Vitamin D significantly reduced high-sensitivity (hs)-CRP at 1600 IU/day in overweight pregnant women or those with GDM [86], and at 400 IU/day in GDM women [87]. In a four-arm trial in GDM, 50,000 IU every two weeks or monthly lowered insulin, HOMA-IR, and the change in total cholesterol compared with placebo, whereas hs-CRP, fasting glucose, and triglycerides did not differ [88]. TNF-α and IL-6 were significantly reduced at 2000 IU/day, with a 36% increase in regulatory IL-10+ CD4+ T cells at 2000 IU/day vs. 400 IU/day (*p* = 0.007) [58,59,119]. Four studies found no significant CRP change [59,70,90,119], and two reports found no significant changes in TNF-α between groups [86,119]. Changes in lipid and glycemic markers were observed predominantly in GDM or obese subgroups [86,87], with improvements in HDL-C, LDL-C, fasting glucose, HbA1c, and HOMA-IR [70,86,87]. In non-GDM populations, vitamin D showed no significant glycemic effects [57,59,119]. Oxidative stress was evaluated in two studies, in which vitamin D significantly decreased malondialdehyde levels, an indicator of oxidative stress [87].

Maternal clinical outcomes. Preeclampsia and gestational hypertension were evaluated in 12 studies. Four studies that compared doses in higher-risk populations reported fewer cases of preeclampsia or hypertension with the higher dose: 50,000 IU every two weeks vs. placebo (15.7% vs. 30.6%, *p* = 0.036) [65]; 4000 IU/day vs. 400 IU/day in vitamin D-deficient women (1 of 83 vs. 7 of 81, 1.2% vs. 8.6%) [35]. Preeclampsia occurred 3.5-fold more often in the lower dose group (500 IU/day), compared with the high-dose group (4000 IU/day, declining to 2000 IU/day) (24% vs. 6.9%, *p* < 0.01) [102]. These results are in accordance with those reported by Xiaomang et al. [85]. In a retrospective cohort, hypertension incidence decreased across the unsupplemented, 2000 IU/day, and 4000 IU/day groups (20.3%, 11.1%, and 6.6%) [95]. A dose-comparison trial in Pakistan (4000 IU/day or 2000 IU/day vs. 400 IU/day) found no difference in preeclampsia (1.3%, 1.2% and 2.2%) [129]. In normotensive vitamin D-deficient women, supplementation with 1000 IU/day was associated with a within-group decrease in systolic blood pressure (105.8 to 104.6 mmHg, *p* = 0.019), which was not compared with the control group [82]. The large MAVIDOS (Maternal Vitamin D Osteoporosis Study) [93] and DALI (Vitamin D and Lifestyle Intervention for Gestational Diabetes Mellitus Prevention) [107,108] trials found no statistically significant differences in preeclampsia or gestational hypertension. Two other studies showed non-significant differences in blood-pressure reduction in the vitamin D group compared with the control group [41,119].

Results for GDM prevention were predominantly neutral across seven studies. DALI trial [107,108] found no major benefit of 1600 IU/day supplementation for GDM prevention in overweight/obese pregnant women, as did the remaining six studies [35,41,57,77,85,129]. The preterm birth rate was lower with higher doses in three studies [87,95,102], but similar between groups in seven studies [35,41,58,85,103,108,129]. Regarding gestational weight gain in women with GDM, one study reported a significant reduction in BMI and gestational edema score in the vitamin D group (*p* < 0.001) [87], whereas another found no significant difference between groups [107]. Cesarean section rates were largely comparable between the supplemented and control groups [35,41,53,65,85,95,103,107,108,119], with the exception of a 1.7-fold higher rate in the 500 IU/day vitamin D group, compared with the higher dose vitamin D group (48% vs. 27.6%; *p* < 0.05) [102].

Perinatal depression was significantly reduced by the administration of 2000 IU/day during the third trimester (*p* = 0.01 at 38–40 weeks; *p* < 0.001 at 4 and 8 weeks postpartum) [69] but not at 4000 IU/day starting in the first trimester, for 18 weeks (*p* = 0.505) [57]. The two trials differed in dose, timing and instrument, so the discrepancy cannot be attributed to any of these factors. Postpartum hemorrhage was significantly reduced at 1000 IU/day compared with placebo (12% vs. 17%, *p* = 0.01) [94], with a non-significant trend in another study (32.1% vs. 38.1%, *p* = 0.054) [104].

Adverse events were collected in six of the 23 vitamin D studies: three reported no gastrointestinal adverse events [65,70,119], one monitored hypercalcemia and hypercalciuria and found no adverse event related to vitamin D [107], one prespecified and monitored adverse and serious adverse events, hypercalcemia and hypervitaminosis D and observed none [129], and one monitored headache and vomiting monthly but did not report the results [88]. The remaining 17 did not report whether adverse events were collected. One descriptive report documented transient neonatal hypercalcemia (total calcium 3.09 mmol/L; reference 1.9–2.6 mmol/L) in an infant whose mother had taken 4000 IU/day from before conception until delivery at 39 weeks [105], underscoring the importance of dose monitoring.

Fetal and neonatal outcomes. Fetal or neonatal outcomes were evaluated in 17 of the 23 studies, including the case report. Most studies reported no significant effect on birth weight or low birth weight, including MAVIDOS [94,103], DALI [108] and the Pakistani dose-comparison trial [129], as well as other studies [35,41,42,53,68,85,119]. One open-label trial in vitamin D-deficient women reported higher birth weight with 1000 IU/day than with routine care (*p* < 0.001) [82]. Motamed et al. [58] reported a greater birth weight in the 2000 IU/day group than in the 1000 IU/day group (3370 ± 450 g vs. 3103 ± 440 g, *p* = 0.018), and Ku et al. [77] found a lower incidence of low birth weight with 800 IU/day compared with 400 IU/day in obese women (3.9% vs. 17.0%, *p* = 0.032). Apgar scores were not significantly different in most studies [42,53,58,95,107,119], but less favorable distributions were reported in the 500 IU/day group compared with the high-dose group, favoring higher-dose supplementation [102].

Three dose-comparison studies reported lower rates of IUGR with the higher dose: 4000 IU/day vs. 400 IU/day (9.6% vs. 22.2%, *p* = 0.027) [35], a significantly lower IUGR index in the higher-dose group [85], and a 4.5-fold higher rate of IUGR in the 500 IU/day group compared with the 4000 IU/day group (16% vs. 3.45%, *p* < 0.01) [102]. Fetal distress, neonatal hypoglycemia, and asphyxia rates were significantly reduced in one GDM trial (*p* < 0.05) [87]. Congenital anomaly rates did not differ significantly between supplemented and unsupplemented groups in one retrospective cohort (*p* = 0.691) [95].

Vitamin E (*n* = 1)

One RCT (*n* = 299) in primigravidae evaluated vitamin E (tocotrienol-rich fraction, TRF) 100 mg/day from 12–16 weeks of gestation until delivery [72]. Preeclampsia incidence was significantly lower in the TRF group, as was pregnancy-induced hypertension (*p* < 0.05). No significant differences were found for preterm birth, mode of delivery, birth weight, or neonatal adverse events.

#### 3.3.2. Omega-3 Fatty Acids (Algal Oil, Fish Oil, EPA/DHA) (*n* = 17)

Omega-3 fatty acids were evaluated in 17 unique studies (20 publications), making them the second most frequently studied supplement after vitamin D. Studies evaluated DHA and EPA, or combined EPA + DHA (fish oil or algal sources) at 200–2000 mg/day, in healthy women, women at high risk of preterm birth, women with GDM, and women with prior pregnancy loss.

Maternal biochemical outcomes. Anti-inflammatory effects of omega-3 supplementation during pregnancy in women with GDM, obesity, or preterm birth risk were significant in three studies [50,116,118]. Fish oil 1000 mg twice daily for 6 weeks in women with GDM significantly downregulated IL-1 (*p* = 0.007) and TNF-α (*p* = 0.01) gene expression in peripheral blood mononuclear cells and reduced hs-CRP levels compared with placebo (*p* = 0.01); IL-8 gene expression was not influenced [50].

In overweight/obese women, 2000 mg/day EPA/DHA initiated in early pregnancy reduced both systemic and placental inflammation [116]. Higher-dose DHA (1000 mg/day) was associated with better preservation of soluble receptor for advanced glycation end products (sRAGE) levels and a greater rise in IL-6 at delivery. The authors interpreted this as evidence of immunomodulation rather than straightforward inflammation suppression [118]. Four studies reported lower triglycerides levels [43,50,78,84]. Findings for total cholesterol, LDL-C, and HDL-C were divergent. Three studies evaluated the impact of omega-3 supplementation on glucose metabolism, and the effect was modest: fasting plasma glucose was significantly reduced in women with GDM (*p* = 0.02) [50], and cord blood glucose was reduced with 800 mg/day than with 200 mg/day (*p* < 0.05) in overweight/obese women [113], but no effect on oral glucose tolerance test (OGTT) results or insulin resistance was observed in obese non-GDM pregnant women [116].

Maternal clinical outcomes. Preeclampsia and gestational hypertension were not significantly affected in the majority of trials [44,84,109,111,118,124]. Kuper et al. [115] reported no significant effect on pregnancy-associated hypertension overall, although the effect among smokers was favorable. Preterm birth was the primary outcome in 12 studies. Makrides et al. [123] (*n* = 5474; 900 mg/day of n-3 PUFA) found no significant difference in early preterm birth overall (2.2% omega-3 group vs. 2.0% control group, *p* = 0.50). A companion analysis by Simmonds et al. [124] explored baseline omega-3 status as a potential effect modifier. Among women with a baseline omega-3 status ≤4.1% of total fatty acids, supplementation substantially reduced the risk of early preterm birth (0.73% vs. 3.16% with control), whereas among women with a baseline status >4.9%, supplementation was paradoxically associated with an increased risk (2.20% vs. 0.97%), thus identifying the women most likely to benefit from supplementation. Two studies found significantly lower rates of preterm delivery with omega-3 supplementation: one in high-risk pregnancies (18.5% vs. 32.8%, *p* < 0.05) [34] and one in smokers, in which the benefit was attributed to antioxidant countereffects on smoking-related oxidative stress [115]. Monthé-Drèze et al. [116] found a longer gestation in the omega-3 group (2000 mg/day EPA/DHA) compared with placebo (40 vs. 39 weeks, *p* = 0.02). Seven other studies found no significant reduction in preterm birth [44,79,80,84,109,111,118].

Adverse events were reported in seven of the 17 omega-3 studies: serious adverse events were prespecified and monitored in four trials [109,111,113,123], and data on gastrointestinal tolerability was collected without serious-event monitoring in three others [34,61,80]. Makrides et al. [123] recorded serious adverse events in 0.5% vs. 0.4% of mothers and in 5.0% vs. 4.0% of infants or pregnancies (relative risk 1.27, 95% CI 0.99–1.62). Garmendia et al. [113] recorded 238 serious adverse events, mostly maternal hospitalizations, and none attributed to DHA. Carlson et al. [109] recorded fewer maternal (chorioamnionitis, rupture of membranes, pyelonephritis) and neonatal serious adverse events with 1000 mg/day than with 200 mg/day, and Carvajal et al. [111] recorded none. The authors of these trials attributed no serious adverse event to supplementation. The remaining ten omega-3 studies did not report whether adverse events were monitored.

Fetal and neonatal outcomes. Fetal outcomes were assessed in 15 studies, 14 of which evaluated birth weight. Five studies reported a significantly higher birth weight or a lower LBW frequency: an LBW frequency of 7.6% in the fish oil group vs. 0% in the placebo group (*p* = 0.02) [61], a higher birth weight with 450 mg/day DHA (3174 g vs. 2890 g, *p* = 0.018) [114], and a significantly improved normal birth weight for gestational age together with a reduced LBW frequency in high-risk pregnancies [34]. Among smokers, omega-3 supplementation significantly reduced LBW frequency [115], and in overweight/obese women, 2000 mg/day EPA/DHA increased the birthweight-for-gestational-age z-score compared with placebo (*p* = 0.02) [116]. Garmendia et al. [113] reported significant birth weight differences between omega-3 supplemented (800 mg/day DHA) groups who attended all dietary sessions and groups with lower attendance (*p* < 0.005), with no significant differences between groups regarding macrosomia (>4000 g) incidence. Eight studies found no significant differences in birth weight [44,50,80,84,109,111,123,127].

Congenital malformations were assessed in two trials with no significant differences [61,113]. One study (*n* = 48) specifically evaluated fetal ductus arteriosus constriction (a theoretical concern with omega-3 supplementation) with 450 mg/day DHA in the third trimester and found no significant between-group differences in prostaglandin E2 levels or ductal pulsatility index [120].

#### 3.3.3. Probiotics (*n* = 11)

Probiotics were evaluated in 11 unique studies (13 publications), representing the third largest supplement category. Probiotic species included *Lactobacillus rhamnosus*, *Lactobacillus acidophilus*, *Lactobacillus casei*, *Lactobacillus plantarum*, *Lactobacillus delbrueckii bulgaricus*, *Bifidobacterium species*, and multispecies combinations (4 × 10^9^ to 450 × 10^9^ CFU/day). Populations included overweight/obese women, women with GDM, women at high-risk of preterm birth, and healthy pregnant women.

Maternal biochemical outcomes. Three trials reported inflammatory and oxidative-stress outcomes in GDM populations, demonstrating significant probiotic-associated improvements: reductions in IL-6, TNF-α, and hs-CRP with VSL#3 supplementation [47], and lower hs-CRP (*p* < 0.05) and TNF-α (*p* < 0.05), but not IL-6, after 8 weeks of a quadruple-strain probiotic (*L. acidophilus* LA-5, *Bifidobacterium* BB-12, *S. thermophilus* STY-31, *L. delbrueckii bulgaricus* LBY-27), together with lower malondialdehyde and higher glutathione reductase and glutathione peroxidase, while total antioxidant capacity did not change significantly [46]. Six weeks of administration of *Lactobacillus acidophilus*, *Lactobacillus casei*, *Bifidobacterium bifidum*, and *Lactobacillus fermentum* [39] upregulated peroxisome proliferator-activated receptor (PPAR)-gamma (*p* = 0.01), transforming growth factor (TGF)-beta (*p* = 0.002), and vascular endothelial growth factor (VEGF) (*p* = 0.006) gene expression in peripheral mononuclear cells. Three RCTs [39,47,51] in women with GDM reported significant reductions in serum insulin and HOMA-IR compared with placebo. Two of these demonstrated reductions in fasting plasma glucose and an improved quantitative insulin sensitivity index (QUICKI) [39,51], while the high-dose VSL#3 formulation improved only insulin resistance indices, without affecting fasting plasma glucose or HbA1c, suggesting strain- and dose-dependent variation [47]. In a non-GDM population [121] *Lactobacillus rhamnosus* HN001 lower fasting plasma glucose (*p* = 0.040) and conjugated bile acid concentrations; the latter correlated positively with both fasting plasma glucose (*p* = 0.020) and fasting insulin (*p* = 0.036), pointing to a possible bile acid-mediated metabolic mechanism. Two GDM trials [39,51] reported significant reductions in serum triglycerides and VLDL-C (*p* < 0.05). Babadi et al. [39] also observed an increase in HDL-C (*p* = 0.03).

Maternal clinical outcomes. GDM incidence was reported in four probiotic trials. Three found no significant reduction [67,97,121]. Wickens et al. [122,125] reported a trend toward lower incidence by the IADPSG definition (relative risk 0.59, 95% CI 0.32–1.08, *p* = 0.08) and a significantly lower prevalence of GDM by the New Zealand definition (2.1% vs. 6.5%, *p* = 0.03). Preeclampsia was not significantly affected in any study [45,67,97,121]. Significant blood-pressure reductions were observed with synbiotic supplementation in women with mild preeclampsia [60] and with a quadruple-strain probiotic in women with GDM [45], in whom blood pressure was significantly lower than placebo at 6 and 8 weeks of administration (*p* < 0.01). Callaway et al. [121] reported that excessive gestational weight gain was less frequent in the probiotic group than in the placebo group (32.5% vs. 46.0%, *p* = 0.01).

Preterm birth was significantly reduced in the probiotic group in one retrospective cohort of high-risk women (2.2% vs. 25.0%, *p* = 0.001), as was chorioamnionitis (2.2% vs. 15.8%, *p* = 0.030) [76]. No other study found a significant effect on preterm birth [121,125]. Mode of delivery did not differ significantly in five studies [51,67,96,97,125].

Adverse events were collected in two of the 11 probiotic studies. A non-randomized study reported fewer gastrointestinal symptoms (bowel pain, constipation, colitis, meteorism, and flatulence) in the probiotic group [96]. A randomized pilot trial prespecified side effects and serious adverse events, recorded them at baseline and at 8 weeks, and found no difference between groups [91]. Nine studies did not report whether adverse events were collected.

Fetal and neonatal outcomes. Fetal outcomes were assessed in six of the 11 probiotic studies. Birth weight was not significantly affected in most trials [51,67,97,121,125], although it was significantly higher in the trial of women at high risk of preterm birth (2712 g vs. 2375 g, *p* = 0.021) [76]. A significant improvement in the five-minute Apgar score was observed with HN001 supplementation (*p* = 0.04) [125]. No other neonatal parameter, including NICU admission, infant length, ponderal index, head circumference, hypoglycemia, jaundice, or congenital anomalies, differed significantly between groups [51,76,121,125]. Congenital anomalies were compared in one trial, with no difference between groups [121].

#### 3.3.4. Minerals

Zinc (*n* = 6)

Six unique studies (7 publications) evaluated 15–40 mg/day of zinc in pregnant women with GDM or impaired glucose tolerance, women at risk of IUGR, women with a history of preterm premature rupture of membranes (PPROM), women with chronic hypertension, and healthy pregnant women [56,63,64,66,71,75,128].

Maternal biochemical outcomes. Four studies reported lower inflammatory or oxidative-stress markers (IL-6, vaspin, TNF-α, leptin, hs-CRP, malondialdehyde) and higher total antioxidant capacity with zinc, although the markers and populations differed between studies [56,63,64,75].

Zinc supplementation significantly improved insulin sensitivity (reduced HOMA-IR and increased QUICKI, *p* < 0.001), in women at risk of IUGR, without affecting the lipid profile or the uterine artery pulsatility index [56]. In insulin-treated women with GDM, 25 mg/day of zinc sulfate from the third trimester was associated with a smaller increase in insulin dose than placebo (8.8 vs. 17.5 IU, *p* = 0.001) [128]. These findings suggest that zinc’s metabolic benefits may extend beyond high-risk glycemic populations.

Maternal clinical outcomes. Preeclampsia was significantly lower in hypertensive women who received 20 mg/day from the first trimester than in the untreated group (21.2% vs. 38.6%, *p* = 0.01), with no significant effect on preterm birth or cesarean delivery rates [75]. Three studies found no significant effect of 15 mg/day [71], 25 mg/day [128], or 40 mg/day [66] of zinc on preeclampsia, preterm delivery, or PPROM. Mode of delivery was evaluated in four studies, none of which found significant differences between groups [66,71,75,128]. A 3.7% dropout rate due to gastrointestinal symptoms (diarrhea) at 40 mg/day of zinc was noted [64].

Fetal and neonatal outcomes. Birth weight and other neonatal outcomes (neonatal height, head circumference, neonatal length, LBW frequency, macrosomia, Apgar score) did not differ significantly between zinc and comparator groups [64,66,71]. One trial in women with GDM reported fewer macrosomic neonates with zinc (2 of 30 vs. 10 of 30, *p* = 0.01); adverse events were not reported [128].

Magnesium (*n* = 2)

Two unique studies (three publications) evaluated the effects of 300–400 mg/day of magnesium citrate supplementation.

Maternal biochemical outcomes. Bullarbo et al. [92] evaluated the administration of 400 mg/day of magnesium or placebo from the first trimester and observed no significant differences between groups in IL-6 or CRP levels.

Maternal clinical outcomes. The BRAMAG (BRAzil MAGnesium) trial [110,112] included pregnant women at higher risk of preterm birth or adverse perinatal outcomes, who received 300 mg/day of magnesium citrate or placebo from 12 to 20 weeks until delivery; no difference between groups was observed in preeclampsia or severe gestational hypertension. Gastrointestinal symptoms were recorded in only one of the two studies, in which nausea was the main reason for dropout [92].

Fetal and neonatal outcomes. No significant differences between the magnesium (300–400 mg/day) and placebo groups were observed for birth weight, gestational length at birth, SGA, Apgar score, NICU admission, or neonatal death [92,110,112].

#### 3.3.5. Other Substances

Alpha-lipoic acid (ALA) (*n* = 2)

Two unique studies evaluated ALA supplementation in pregnant women with GDM, at 100 mg/day (one trial, 3 publications [36,37,38]) and 300 mg/day [55].

Maternal biochemical outcomes. Both trials reported lower fasting glucose with ALA than with placebo (*p* < 0.001) and a lower malondialdehyde/total antioxidant capacity ratio. The 100 mg/day trial additionally reported lower HOMA-IR and higher QUICKI [37]; lower triglyceride-related indices (TG, TG/HDL-C, atherogenic index of plasma (AIP) and triglyceride-glucose index; *p* < 0.01) without change in total cholesterol or LDL-C; lower thiobarbituric acid-reactive substances (TBARS), a marker of lipid peroxidation (*p* < 0.001) [38]; and lower gamma-glutamyl transferase [36]. HbA1c did not change significantly (*p* = 0.059) [38]. 

Maternal clinical outcomes. No preterm births occurred in either group of the 100 mg/day trial [36,37,38], and systolic blood pressure did not differ between groups [38]. Neither trial reported whether adverse events were collected.

Fetal and neonatal outcomes. Fetal and neonatal outcomes were assessed only in the 300 mg/day trial, which found no significant differences in birth weight, length or head circumference between ALA and placebo [55].

Curcumin (*n* = 1)

A single 100 mg oral dose in women with preeclampsia scheduled for cesarean section showed no significant differences in serum COX-2 or IL-10 levels. No safety outcomes were reported beyond inflammatory markers [74].

Ginger (*n* = 2)

Two unique studies [40,100] evaluated the effects of ginger supplementation during pregnancy: one RCT in women with GDM and one uncontrolled feasibility study in women with gastrointestinal discomfort. The RCT (1000 mg/day ginger rhizome capsules for 8 weeks) reported no significant differences between groups in fasting plasma glucose or HbA1c, suggesting that the proposed anti-inflammatory and insulin-sensitizing mechanisms did not translate into a measurable glycemic benefit. Adverse events were limited to mild gastrointestinal symptoms (stomachache and flatulence) [40]. Laekeman et al. [100] conducted an uncontrolled prospective feasibility study of a concentrated ginger root extract (maximum 100 mg/day, taken as needed) in 51 women with gastrointestinal discomfort, of whom 44 took the extract (1 to 55 tablets). They reported two pregnancy losses (one at 17 weeks in a woman who had taken two tablets and one in a woman who took no extract), ten births before 37 weeks (nine of the 44 exposed and one of the six unexposed women), four cases of developmental dysplasia of the hip and two minor malformations. None of these events were related to the number of tablets taken. Without a comparator, no inference about the extract can be drawn in either direction.

Epigallocatechin-3-gallate (EGCG) (*n* = 2)

Two unique studies [81,89] evaluated purified EGCG (≥95% and ≥98%, Sigma-Aldrich) supplementation. As an adjunct to nifedipine in severe preeclampsia, EGCG 100 mg/dose (up to five doses) significantly reduced blood-pressure control time (31.2 ± 16.7 min vs. 45.3 ± 21.9 min, *p* = 0.03) and prolonged the interval to the next hypertensive crisis (7.2 ± 2.9 h vs. 4.1 ± 3.7 h, *p* = 0.02). No severe maternal adverse events occurred, and mild gastrointestinal symptoms (nausea, vomiting, dizziness) were similar between groups [81]. In GDM, EGCG 500 mg/day was associated with a lower LBW (<2600 g) incidence (*p* = 0.03), higher Apgar scores at 1 and 5 min (*p* < 0.05) and a lower incidence of neonatal hypoglycemia (*p* = 0.01) [89].

Resveratrol (*n* = 2)

Two unique studies [73,101] evaluated resveratrol, either as an adjunct to nifedipine in pregnant women with severe preeclampsia or combined with inositols in overweight pregnant women. Trans-resveratrol (80 mg/day) combined with inositols significantly reduced fasting plasma glucose, total cholesterol, LDL-C and triglycerides versus placebo at 30 and 60 days [101]. Neither study found significant differences in blood pressure, clinical adverse events, birth weight or Apgar scores [73,101].

Chlorella (*n* = 1)

Chlorella at 6000 mg/day significantly reduced constipation (*p* < 0.05) in pregnant women with low-grade inflammation [83]. Two participants in the control group withdrew due to severe pregnancy-induced hypertension and eclampsia. Birth weight, Apgar scores and other neonatal anthropometric measures did not differ significantly between groups.

#### 3.3.6. Combination Supplements (*n* = 10)

Ten unique studies (12 publications) [48,49,52,54,60,62,98,99,104,106,117,126] evaluated combinations of two or more supplements: synbiotics (probiotic with prebiotic); omega-3 fatty acids co-supplemented with vitamin E, vitamin D, or probiotics; magnesium combined with vitamin E; a nutraceutical complex of omega-3/anthocyanins/alpha-cyclodextrins; and the combination of antioxidant vitamins C and E, predominantly in populations with GDM or preeclampsia.

Maternal biochemical outcomes. Inflammatory biomarkers (hs-CRP) were evaluated in six studies [48,52,62,98,99,106,117]. Significant reductions in hs-CRP were observed following synbiotic supplementation (*p* = 0.004) [52] and combined vitamin D plus omega-3 (*p* = 0.008) [62], but not with other combinations [98,99]. Combined vitamin D (50,000 IU every two weeks) plus omega-3 (1000 mg/day), as well as magnesium oxide (250 mg/day) plus vitamin E (400 IU/day), showed significant reductions in triglycerides, VLDL-C, fasting plasma glucose, serum insulin, and HOMA-IR compared with the individual supplements and with placebo [49,54]. In contrast, neither fish oil combined with probiotics in overweight/obese pregnant women [104] nor the combination of omega-3 fatty acids, anthocyanins and alpha-cyclodextrins [106] in women with GDM changed fasting plasma glucose or insulin concentrations significantly. A double-blind, placebo-controlled RCT [117] in 98 pregnant women with obesity (BMI ≥ 30 kg/m^2^) administered vitamin E (30 α-tocopherol equivalents/day) with vitamin C (90 mg/day), vitamin B6 (18 mg/day) and folic acid (800 µg/day) in addition to a standard prenatal vitamin from before 13 weeks of gestation until delivery; no significant changes were observed in CRP, IL-6, IL-8, IL-1β or oxidative-stress markers.

Maternal clinical outcomes. Preeclampsia was not significantly affected in three combination supplement trials (vitamin D plus omega-3, fish oil plus probiotic, and synbiotic) [52,62,104]. A significantly lower rate of preterm births (<37 weeks) was observed in the vitamin C and E supplementation group compared with the untreated group (12% vs. 22%, *p* < 0.05) [126]. In the same trial, pregnant women in the control group were 1.26 times more likely to undergo lower-segment cesarean section than those receiving vitamins C and E. In another vitamin C and E supplementation study [117], the incidence of GDM, pregnancy hypertension, cesarean section, and preterm birth did not differ from the control group. Karamali et al. [52] reported a significantly lower cesarean section rate in the synbiotic group compared with placebo (*p* = 0.04).

Adverse events were collected in two of the 10 combination studies: fish oil capsules caused adverse effects in 28% of women in one trial [104], and minor gastrointestinal symptoms were balanced between arms in the other [117]. The remaining eight did not report whether adverse events were collected.

Fetal and neonatal outcomes. Birth weight, macrosomia, neonatal length, head circumference, Apgar scores, SGA rates, and congenital malformations did not differ significantly in any combination trial that reported them [52,62,104,106,117,126].

### 3.4. Evidence Gap Map

**Figure 2:**
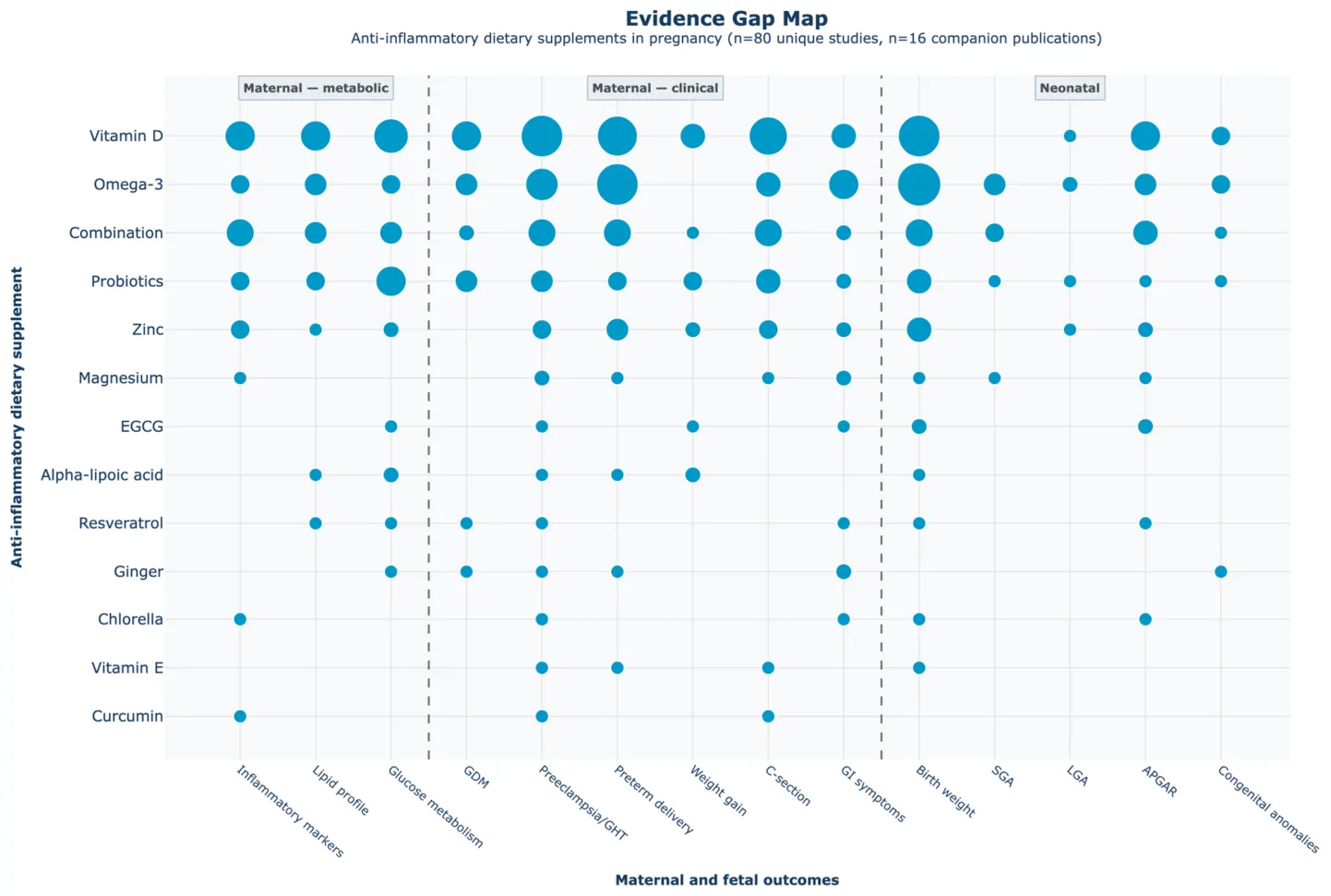
Evidence gap map for anti-inflammatory dietary supplements used in pregnancy. Each circle marks an outcome domain reported by at least one publication for that supplement: circle area is proportional to the number of publications reporting the outcome. An empty cell means that no included publication reported the outcome. Circle size reflects the volume of reporting, not the direction, size or quality of any effect. (GDM = gestational diabetes mellitus; GHT = gestational hypertension; GI = gastrointestinal; SGA = small for gestational age; LGA = large for gestational age).

The evidence gap map (Figure 2) summarizes the distribution of 96 publications (80 individual studies and 16 companion publications of 12 families) by supplement type and outcome domain, highlighting the real gaps in this field.

Anti-inflammatory supplements (vitamin D, omega-3, probiotics) dominate the upper half of the evidence gap map, while the lower half remains nearly empty. Minor supplements are poorly represented: curcumin, vitamin E, and chlorella by a single study each, and ginger, epigallocatechin-3-gallate (EGCG), resveratrol, magnesium and alpha-lipoic acid by two studies each. Together they contribute sparse data on inflammatory markers, preeclampsia, mode of delivery, and birth weight. Maternal biochemical and clinical outcomes (GDM, preeclampsia, preterm birth) are relatively densely covered, reflecting the focus on gestational diabetes, obesity, and high-risk populations. Neonatal outcomes are sparsely covered: SGA, LGA, and congenital anomalies are among the emptiest cells on the map, and Apgar score data, although present for vitamin D and omega-3, are absent for most other supplements.

The initiation of anti-inflammatory dietary supplements was more frequent in the second and third trimesters or was recorded without a specified gestational period in the retrospective cohorts [76,78,84]. First-trimester supplementation (the period of organogenesis and the most relevant for congenital anomalies) occurred in only 19 of the 80 studies, and in a further six, the initiation window included the first trimester (Table 1). This is important to mention because these two factors are interrelated: the outcomes for which data on early exposure are most needed are also those for which the least data are available. The resulting gaps in congenital anomaly and early pregnancy outcome data reflect structural barriers (ethical constraints on early recruitment, dropout due to early miscarriage) [130] rather than missing results.

## 4. Discussion

### 4.1. Summary of Principal Findings

This scoping review mapped the published clinical evidence on anti-inflammatory dietary supplements during pregnancy, based on 80 unique studies. Two dietary supplements, vitamin D (23 studies) and omega-3 fatty acids (17 studies), account for half of the entire literature, followed by probiotics (11 studies) and combination formulations (10 studies).

For vitamin D, adverse events were collected in six of the 23 studies: none reported gastrointestinal adverse events, two trials that monitored hypercalcemia or serious adverse events observed none [107,129], and one monitored symptoms without reporting them [88]. The other 17 did not report whether adverse events were collected. One case report described transient neonatal hypercalcemia after maternal intake of 4000 IU/day [105], from before conception until delivery at 39 weeks, a finding that underscores the need for dose monitoring rather than indicating a general hazard. Three dose-comparison studies in at-risk populations reported fewer cases of preeclampsia, hypertension or intrauterine growth restriction (IUGR) with higher doses [85,95,102], and a fourth dose-comparison trial found no difference [129]. In contrast, the two largest placebo-controlled trials reported no significant differences [93,107,108]. These studies vary in design, dosage, and population and were not critically appraised, so no definitive conclusions regarding dose–response can be drawn. Results for GDM prevention were predominantly neutral, and those for preterm birth were mixed.

Serious adverse events were prespecified and monitored in six studies: four omega-3 trials [109,111,113,123], one probiotic pilot trial [91], and one vitamin D dose-comparison trial [129]. One small trial found no change in fetal ductus arteriosus flow with 450 mg/day of DHA [120], addressing the theoretical concern of ductus arteriosus constriction.

In the two probiotic studies that collected adverse events, gastrointestinal symptoms were no more frequent with probiotics than with placebo [91,96]. One trial additionally reported no difference in congenital anomalies [121]. The omega-3 and probiotic trials include the largest and most completely reported studies in the map [131].

The remaining anti-inflammatory dietary supplements with documented anti-inflammatory mechanisms, such as curcumin, resveratrol, epigallocatechin-3-gallate (EGCG) and ginger, have been covered by only one or two studies. This concentration of studies on a small number of dietary supplements, along with the lack of information and the absence of controlled clinical studies on herbal supplements, is the defining characteristic of this field.

Uncertain or limited evidence. Zinc supplementation was associated with lower inflammatory and oxidative-stress markers in four trials and a lower preeclampsia incidence in one retrospective cohort of hypertensive women, but with no difference in obstetric outcomes in low-risk or PPROM populations [56,63,64,75]; 3.7% of participants dropped out because of gastrointestinal symptoms at 40 mg/day [64].

Magnesium [92,110,112] showed no significant biochemical or clinical effects. Gastrointestinal symptoms were recorded in one trial, in which nausea was the main reason for dropout [92]. Alpha-lipoic acid was studied in two trials in GDM [36,37,38,55], both reporting lower fasting glucose and oxidative-stress markers; neither reported adverse events, and fetal outcomes were assessed in only one trial [55]. Results for combination dietary supplements were inconsistent, with lower hs-CRP and glycemic markers in some co-supplementation trials [48,52,54,62] and no significant effects in others [98,99,104,106,117].

Absence of controlled safety data. For herbal supplements, no included study collected adverse events systematically. Curcumin was evaluated in one trial with a single 100 mg dose, with no outcomes beyond inflammatory markers [74], consistent with the previously reported absence of RCT evidence in pregnant women [132]. Chlorella [83], vitamin E [72], EGCG [81,89], and resveratrol [73,101] provide isolated observations, several of them as adjuncts to drug treatment. For ginger, the only fetal data come from an uncontrolled feasibility study in which pregnancy losses, preterm births, and hip dysplasia occurred at frequencies that cannot be compared with any reference group [100]. This mirrors the broader literature, in which teratogenicity, pharmacokinetic, and long-term child outcome data are absent for most anti-inflammatory herbal supplements [132,133,134,135].

### 4.2. Comparison with Existing Literature

Our findings are concordant with previously reported data. Omega-3 fatty acids are among the most studied anti-inflammatory dietary supplements in pregnancy, and reductions in pro-inflammatory and oxidative-stress markers have been reported in meta-analyses, particularly in GDM [131]. The maternal adverse events recorded in the trials that collected them (minor gastrointestinal symptoms) align with published data [136,137], and the modulation of maternal inflammatory responses by higher doses of DHA [118] is consistent with the proposed mechanism for the reduction in preterm birth risk.

Ginger is the most investigated herbal supplement in pregnancy for nausea and vomiting, with a well characterized anti-inflammatory effect mediated by inhibition of prostaglandin synthesis and the NF-kB pathway [138]. However, its efficacy in pregnancy-related inflammatory complications (preeclampsia and GDM) has rarely been studied [139]. Our scoping review confirms this finding: only one study addressed GDM, and the only fetal data is derived from an uncontrolled feasibility study that could not test any relationship between the extract and pregnancy complications or malformations.

Curcumin has attracted substantial preclinical interest because of its anti-inflammatory properties, acting by inhibiting NF-kB pathway and reducing pro-inflammatory cytokines (TNF-α, IL-6, IL-1β), with animal and in vitro evidence of efficacy in preeclampsia, preterm birth, and GDM [132,140,141]. The lack of clinical evidence for curcumin aligns with the previous reports of the gap between preclinical promise and clinical evidence for this active compound [132]. Similarly, the reliance on animal and in vitro data for polyphenols in the scientific literature [140,141,142] is reflected in our scoping review, where resveratrol and EGCG appear only in small adjunctive trials rather than as primary anti-inflammatory or antioxidant interventions. The defining characteristics of this field remain the concentration of evidence on a small number of well-studied supplements, the absence of controlled safety data for herbal compounds, and the consistent underrepresentation of first-trimester exposure and neonatal endpoints, precisely the areas in which adequately powered clinical trials are most needed.

### 4.3. Implications

Vitamin D, omega-3 fatty acids, and probiotics are the supplements with the most trials in the map. Serious adverse events were monitored in six studies, four of which were omega-3 trials. The evidence is thinner than the trial count suggests: two-thirds of the studies did not report whether adverse events were collected, three-quarters did not initiate supplementation in the first trimester, and only two publications followed infants beyond the neonatal period, to 8 weeks [68] and approximatively 3 months [114].

For zinc, magnesium, alpha-lipoic acid, and combination regimens, the available evidence was derived from two to ten studies each. For curcumin, chlorella, vitamin E, EGCG, resveratrol, and ginger, the map includes only one or two studies per supplement, several of them adjuncts to drug therapy, and no controlled safety data. There is a descriptive report of a neonatal signal (hypercalcemia following high-dose maternal vitamin D [105]) and an uncontrolled series of adverse pregnancy outcomes among women taking ginger extract [100], which cannot be directly attributed to the supplement. Consequently, the map does not provide support for claims regarding the safety of these supplements during pregnancy. For the anti-inflammatory dietary supplements lacking controlled pregnancy data, absence of evidence is not evidence of safety—a principle especially relevant to teratogenicity and first-trimester exposure [133,134]. Pregnant women should be advised that “natural” does not imply “safe”, an assumption that scientific literature identifies as the primary reason for use [133,134,143].

### 4.4. Strengths

We conducted a broad, cross-database search strategy that captured both the efficacy-oriented RCT literature and sparser observational and case-report evidence, enabling primary trials to be distinguished from companion publications (96 publications reduced to 80 unique studies, linked by trial registration number and setting as documented in Supplementary Materials, Table S3) and preventing the double-counting of shared cohorts.

This scoping review covers a comprehensive range of supplements, mapping 12 anti-inflammatory dietary supplements across unique trials, encompassing up to 19 distinct agents once the individual components of combination regimens are counted (synbiotics; omega-3 with vitamin E, vitamin D or probiotics; magnesium with vitamin E; omega-3/anthocyanins/alpha-cyclodextrins; vitamin C with vitamin E), allowing the striking imbalance between well-studied compounds and evidence-poor herbal supplements to be characterized directly.

### 4.5. Limitations

Limitations of the evidence base. First, the evidence is highly concentrated: vitamin D, omega-3 fatty acids and probiotics account for the majority of large trials, while eight dietary supplements are supported by only one or two studies each (curcumin, chlorella and vitamin E by a single study). Second, populations were often selected (GDM, overweight/obesity, preeclampsia, high risk of preterm birth), which limits the extrapolation of dietary supplement use in healthy pregnancies, and studies were concentrated in West Asia, which limits the generalizability of population characteristics and co-interventions. Third, the window of exposure is narrow, and the period of organogenesis is the least characterized window. Fourth, outcomes are dominated by maternal biochemical markers, with clinical maternal and neonatal outcomes reported as secondary endpoints. Fifth, adverse-event collection was uneven: serious adverse events were prespecified and monitored in six studies (four omega-3 trials, one probiotic pilot trial and one vitamin D trial), adverse events or tolerability data were collected in 20 others, and 54 of the 80 studies did not report whether adverse events were collected; therefore, for most studies, the absence of reported harm reflects the absence of collection; the tolerability described for vitamin D, omega-3 fatty acids, and probiotics rests on this minority of studies. Finally, formulations, doses and timing of administration were heterogeneous, reflecting the lack of standardization described in the literature [134,143], which limited comparability across studies.

Limitations of the review. As a scoping review, the aim was to map the extent and nature of the evidence rather than to appraise it quantitatively. Accordingly, no formal risk-of-bias assessment or meta-analysis was performed; every result reported is the source’s own, unappraised result; and because small trials with biochemical primary outcomes predominate, the findings should not be read as effect estimates. As prespecified in the protocol, the search was restricted to three bibliographic databases. The gray literature and trial registries were not searched, so unpublished studies with null or negative safety findings may be under-represented. The database searches have some limitations. First, the PubMed strategy was corrected and repeated on 14 October 2026 because errors were identified in the strategy executed on 5 June 2026; the corrected search, run with the same filters and the original date window (1 January 2016 to 1 June 2026), retrieved 2280 records, of which 164 had not been screened before, and one additional study was included. Second, the June Scopus and Web of Science strategies applied document-type, subject-area and term-exclusion limits that were not prespecified and could have removed eligible studies that mentioned routine supplements such as iron, calcium or folic acid. Both databases were searched again in October 2026 over the same date window without the subject-area limits and without the micronutrient exclusion terms. The searches retrieved 3179 (Scopus) and 4087 (Web of Science) records, of which 2252 had not been screened before; seven reports were sought in full text and four additional studies were included (three vitamin D trials and one zinc trial), none of which altered the distribution of the map. The second search did not retrieve 248 records from Scopus and 10 from Web of Science, retrieved in June, including one included study. All searches were retained at the identification step, and the records were deduplicated across searches. Case reports published in sources not indexed as articles, letters or notes may still have been missed, which matters for rare adverse outcomes.

### 4.6. Future Research Priorities

The gaps identified in this map highlight the need for further research. First, trials involving vitamin D, omega-3 fatty acids, and probiotics should explicitly define adverse events and systematically collect data on these events for mothers, fetuses, and newborns, including serious incidents, with denominators reported for each group. At present, only a small minority of studies in these three categories adhere to this practice.

Second, fewer than a quarter of the included studies initiated supplementation in the first trimester in all participants (less than a third in any participant), and only eight investigated congenital anomalies. Because randomized trials generally recruit participants after the first trimester, they are unable to assess teratogenic risk. This issue necessitates the use of pregnancy registries and cohorts that enroll women early in pregnancy.

Third, only two publications followed infants beyond the neonatal period (to 8 weeks and to approximatively 3 months of age), and the one parent trial with a 12-month infant outcome reported it outside the included publications. Linking trial cohorts to child health records would allow long-term outcomes to be examined. 

Fourth, meaningful trials of curcumin, EGCG, resveratrol, ginger, chlorella, and vitamin E require standardized formulations with a specified active-compound content. Fifth, a significant portion of the evidence is derived from a single region, with most studies involving women with gestational diabetes or other specific conditions. Consequently, research involving unselected pregnant populations and conducted in diverse regions is necessary to generalize the findings.

Finally, it is crucial to differentiate between tolerability and safety, as well as between biochemical changes and clinical benefits. Such distinctions would allow future evidence maps to clearly separate measured outcomes from those that have been conclusively demonstrated.

## 5. Conclusions

This scoping review mapped 80 unique studies (96 publications) of anti-inflammatory dietary supplements in pregnancy. Three supplements (vitamin D, omega-3 fatty acids and probiotics) together accounted for the majority of studies, whereas several other categories were each represented by only one or two studies, some of which examined the supplement as an adjunct to pharmacological treatment. Most of the included studies were randomized trials with biochemical primary endpoints, conducted in women with gestational diabetes mellitus or other selected conditions, and a large share of the evidence originated from a single geographical region, West Asia. Reported outcomes were dominated by maternal biochemical markers, and clinical maternal and fetal outcomes were mostly secondary outcomes. The map draws a clear distinction between tolerability and safety. Adverse events were collected in approximately a third of the studies, and serious adverse events were prespecified and monitored in six, four of which were omega-3 trials, in which events occurred at comparable rates in both arms and none were attributed to the supplement. Supplementation was initiated in the first trimester in only a minority of studies, leaving the period of organogenesis the least characterized window of exposure.

This scoping review did not evaluate the quality of the studies, and its findings should not be interpreted as effect estimates or as a basis for recommending or discouraging any supplement. Instead, it illustrates the distribution of evidence: it is dense for biochemical outcomes in a few compounds, sparse for clinical and neonatal outcomes, and largely absent for early pregnancy exposure and long-term follow-up across nearly the entire field.

## Figures and Tables

**Table 1:** Gestational timing of supplementation in the included studies, by supplement category (*n* = 80 unique studies).

Supplement Category (Studies, *n*)	T1	T2	T3	>1 Trimester/Not Specified ^1^	Populations Studied	Clinical Outcomes Targeted and Gestational Window in Which They Arise
Vitamin D (23)	7	11	2	3	Healthy pregnancies; GDM; vitamin D deficiency or insufficiency; overweight/obesity; previous or at-risk preeclampsia	Preeclampsia/gestational hypertension (after 20 wk); GDM (screened 24–28 wk); glycemic and inflammatory markers; birth weight and neonatal anthropometry; postpartum hemorrhage; perinatal depression (T3 to 8 wk postpartum); stillbirth
Omega-3 fatty acids (17)	4	9	1	3	Healthy pregnancies; high risk of, or threatened, preterm birth; GDM; overweight/obesity;	Preterm birth (<37 wk) and early preterm birth (<34 wk); GDM; lipid and inflammatory markers; birth weight
Probiotics (11)	2	7	1	1	Healthy pregnancies; GDM; overweight/obesity; high risk of preterm birth; depressive symptoms;	GDM and glycemic markers (24–28 wk); blood pressure; preterm birth and chorioamnionitis; premature rupture of membranes
Combination regimens (10)	2	6	0	2	Healthy pregnancies; GDM; overweight/obesity; mild preeclampsia	Glycemic, lipid and inflammatory markers; GDM; preeclampsia and blood pressure (after 20–24 wk); preterm birth
Zinc (6)	1	4	1	0	Healthy pregnancies; history of PPROM; impaired glucose tolerance; GDM on insulin; risk of IUGR; chronic hypertension	Birth weight and macrosomia; PPROM/PROM; inflammatory and glycemic markers; insulin requirement; preeclampsia (after 20 wk)
Magnesium (2)	2	0	0	0	Healthy pregnancies; high risk of adverse perinatal outcome	Blood pressure rise and preeclampsia
Alpha-lipoic acid (2)	0	2	0	0	GDM	Glycemic, lipid and oxidative-stress markers (after GDM diagnosis, 24–28 wk)
Ginger (2)	1	1	0	0	GDM; gastrointestinal discomfort (uncontrolled feasibility study)	Glycemic control (T2); feasibility and congenital malformations after T1 exposure
EGCG (2)	0	0	2	0	GDM; severe preeclampsia (adjunct to nifedipine)	Maternal glycemic control and neonatal outcomes (T3); blood-pressure control in hypertensive crisis (T3)
Resveratrol (2)	0	1	1	0	Overweight; severe preeclampsia (adjunct to nifedipine)	Metabolic markers (T2); blood-pressure control in hypertensive crisis (T3)
Vitamin E (1)	0	0	0	1	Primigravidae (started at 12–16 wk)	Preeclampsia and pregnancy-induced hypertension (after 20 wk)
Curcumin (1)	0	0	1	0	Preeclampsia at cesarean section (single peri-operative dose)	Inflammatory markers (COX-2, IL-10) at delivery
Chlorella (1)	0	1	0	0	Low-grade inflammation	Constipation; inflammatory markers; neonatal anthropometry
Total (80)	19	42	9	10		

Counts refer to unique studies (companion publications counted once). T1, T2, T3 = number of studies in which supplementation was initiated in the first, second or third trimester, as reported by the study authors. ^1^ Initiation window spanning more than one trimester with no single start point reported, or initiation not specified (retrospective cohorts); this includes one case report with exposure from before conception. Gestational windows in the last column refer to when the targeted outcome is diagnosed or occurs, not to a recommended time of supplementation. The table describes the stages of pregnancy in which each supplement has been studied; it does not indicate when supplementation is indicated or safe. GDM = gestational diabetes mellitus; PPROM = preterm premature rupture of membranes; IUGR = intrauterine growth restriction; EGCG = epigallocatechin-3-gallate; COX-2 = cyclooxygenase-2; IL = interleukin; wk = weeks of gestation.

## Data Availability

The data presented in this study are available in the article and its Supplementary Materials.

## Supplementary Materials

The following supporting information can be downloaded at https://www.mdpi.com/article/10.3390/nu18213627/s1. Table S1. Inclusion and exclusion criteria; Table S2. Search Log in PubMed, Scopus and Web of Science; Table S3. Characteristics of included studies (*n* = 80 unique studies, 96 publications).
