# Supplementary material for: Anti-Inflammatory Dietary Supplements in Pregnancy: A Scoping Review
Source: Nutrients. 2026 Oct 31;18(21):3627. doi: 10.3390/nu18213627 (PMC13765241; doi:10.3390/nu18213627)
Supplement: Supplementary file 1 [file nutrients-18-03627-s001.zip › nutrients-4630866-supplementary.pdf]

Supplementary material

Anti-inflammatory dietary supplements in pregnancy: a scoping review

Authors: Anamaria Apan1†, Cristina Pop1†, Irina Iaru1*, Oliviu Voștinaru1, Răzvan Mircea Cherecheș2, Maria Bonea3, Cristina Mogoșan1
1 Department of Pharmacology, Physiology, Pathophysiology, Faculty of Pharmacy, "Iuliu Hațieganu" University of Medicine and Pharmacy, 400012 Cluj-Napoca, Romania
2 HIVE Health Innovation Center, Babeș-Bolyai University, 400095 Cluj-Napoca, Romania
3 Department of Neurosciences - Psychiatry and pediatric psychiatry, "Iuliu Hațieganu" University of Medicine and Pharmacy, Cluj-Napoca, 400012, Romania
*Correspondence: Irina Iaru; cazacu.irina@umfcluj.ro
†These authors contributed equally to this work.

Table S1. Inclusion and exclusion criteria

PICOS	Inclusion	Exclusion
Population	Pregnant women aged 18+ regardless of their gestational diabetes mellitus (GDM) status.	Non-pregnant population; studies on pregnant women with GDM focusing only on glycemic outcomes without reporting other maternal/fetal.
Intervention	Orally administration of a dietary supplement with anti-inflammatory activity: vitamins (vitamin D, vitamin E), minerals (zinc, magnesium), omega-3 fatty acids (fish oil, EPA/DHA), herbal supplements (Curcumin, Ginger, EGCG, Boswellia, Berberine), probiotics, other substances (resveratrol, coenzyme Q10, quercetin, N-acetylcysteine, bromelain, alpha-lipoic acid, spirulina, chlorella, astaxanthin).	Prescription-only or over the counter medication (e.g. non-steroidal anti-inflammatory drugs); non-oral routes (e.g. intramuscular); food based interventions.
Comparator	Any (active treatment, placebo, no treatment); descriptive designs without a comparator (case reports, case series, single-arm studies)	-
Outcome	Maternal outcomes: adverse events (gastrointestinal symptoms, allergic reactions, bleeding, etc.), pregnancy loss (miscarriage, stillbirth), preterm birth (<37 weeks), preeclampsia/gestational hypertension, gestational diabetes mellitus, prenatal depression/anxiety, gestational weight gain, mode of delivery (c-section rates), changes in inflammatory biomarkers (CRP, IL-6, TNF-α, IL-10), glycemic biomarkers (fasting plasma glucose, HbA1c, insulin resistance, etc.), lipidic profile biomarkers (total cholesterol, LDL-C, HDL-C, triglycerides), and other blood markers (adiponectin, etc).<br>Neonatal outcomes: congenital malformations, birth weight (low birth weight, small/large for gestational age SGA/LGA), Apgar scores, NICU admission, neonatal mortality.	No maternal or neonatal outcomes.
Study design	Randomised clinical trials (RCTs), quasi-experimental, prospective cohort, retrospective cohort, controlled studies, case-control, case-series, case-reports	Reviews, systematic reviews, editorials, conference abstracts, case reports without outcome data; in vitro studies or experimental studies on animal models of pregnancy;

EPA=eicosapentaenoic acid; DHA=docosahexaenoic acid; EGCG = epigallocatechin-3-gallate;CRP=C-reactive protein; IL=interleukin; TNF-α= tumor necrosis factor-α; HbA1c=glycated haemoglobin; LDL-C= low density lipoprotein-cholesterol; HDL-C=high density lipoprotein-cholesterol; NICU=neonatal intensive care unit.

Table S2. Search Log in PubMed, Scopus and Web of Science

Database	String	Total hits
**PubMed**<br>Corrected search, 14 October 2026	("Pregnancy"[MeSH Terms] OR "Pregnant People"[MeSH Terms] OR "Prenatal Care"[MeSH Terms:noexp] OR "Maternal Exposure"[MeSH Terms] OR "Pregnancy Trimesters"[MeSH Terms:noexp] OR "pregnan*"[Title/Abstract] OR "antenatal"[Title/Abstract] OR "prenatal"[Title/Abstract] OR "gestation*"[Title/Abstract] OR "maternal"[Title/Abstract] OR "trimester*"[Title/Abstract] OR "peripartum"[Title/Abstract] OR "intrapartum"[Title/Abstract]) AND ("Dietary Supplements"[MeSH Terms:noexp] OR "vitamin d"[MeSH Terms] OR "zinc"[MeSH Terms] OR "magnesium"[MeSH Terms] OR "Cholecalciferol"[MeSH Terms] OR "Ergocalciferols"[MeSH Terms] OR "vitamin e"[MeSH Terms] OR "alpha-Tocopherol"[MeSH Terms] OR "Tocopherols"[MeSH Terms] OR "fatty acids, omega 3"[MeSH Terms:noexp] OR "Fish Oils"[MeSH Terms] OR "Eicosapentaenoic Acid"[MeSH Terms] OR "Docosahexaenoic Acids"[MeSH Terms] OR "Curcumin"[MeSH Terms] OR "Zingiber officinale"[MeSH Terms] OR "Camellia sinensis"[MeSH Terms] OR "Tea"[MeSH Terms:noexp] OR "Catechin"[MeSH Terms] OR "Berberine"[MeSH Terms] OR "Resveratrol"[MeSH Terms] OR "Quercetin"[MeSH Terms] OR "Acetylcysteine"[MeSH Terms] OR "Bromelains"[MeSH Terms] OR "Thioctic Acid"[MeSH Terms] OR "Spirulina"[MeSH Terms] OR "Probiotics"[MeSH Terms:noexp] OR "Ubiquinone"[MeSH Terms:noexp] OR "vitamin d"[Title/Abstract] OR "Cholecalciferol"[Title/Abstract] OR "calcitriol"[Title/Abstract] OR "ergocalciferol"[Title/Abstract] OR "vitamin e"[Title/Abstract] OR "tocopherol*"[Title/Abstract] OR "omega-3"[Title/Abstract] OR "omega-3"[Title/Abstract] OR "fish oil*"[Title/Abstract] OR "docosahexaenoic"[Title/Abstract] OR "eicosapentaenoic"[Title/Abstract] OR "Curcumin"[Title/Abstract] OR "turmeric"[Title/Abstract] OR "curcuma"[Title/Abstract] OR "ginger"[Title/Abstract] OR "Zingiber"[Title/Abstract] OR "green tea"[Title/Abstract] OR "EGCG"[Title/Abstract] OR "epigallocatechin"[Title/Abstract] OR "Boswellia"[Title/Abstract] OR "frankincense"[Title/Abstract] OR "boswellic acid*"[Title/Abstract] OR "Berberine"[Title/Abstract] OR "Resveratrol"[Title/Abstract] OR "Quercetin"[Title/Abstract] OR "N-acetylcysteine"[Title/Abstract] OR "N-acetyl cysteine"[Title/Abstract] OR "Acetylcysteine"[Title/Abstract] OR "bromelain*"[Title/Abstract] OR "alpha-lipoic acid"[Title/Abstract] OR "alpha-lipoic acid"[Title/Abstract] OR "lipoic acid"[Title/Abstract] OR "Thioctic Acid"[Title/Abstract] OR "Spirulina"[Title/Abstract] OR "Arthrospira"[Title/Abstract] OR "astaxanthin"[Title/Abstract] OR "probiotic*"[Title/Abstract] OR "coenzyme Q10"[Title/Abstract] OR "CoQ10"[Title/Abstract] OR "Ubiquinone"[Title/Abstract] OR "ubiquinol"[Title/Abstract] OR "dietary supplement*"[Title/Abstract] OR "herbal supplement*"[Title/Abstract] OR "nutraceutical*"[Title/Abstract]) AND ("Pregnancy Outcome"[MeSH Terms:noexp] OR "Pregnancy Complications"[MeSH Terms:noexp] OR "pre eclampsia"[MeSH Terms] OR "Eclampsia"[MeSH Terms] OR "abortion, spontaneous"[MeSH Terms] OR "Premature Birth"[MeSH Terms] OR "Fetal Death"[MeSH Terms] OR "Stillbirth"[MeSH Terms] OR "infant, low birth weight"[MeSH Terms] OR "Fetal Growth Retardation"[MeSH Terms] OR "Congenital Abnormalities"[MeSH Terms:noexp] OR "Postpartum Hemorrhage"[MeSH Terms] OR "Maternal Mortality"[MeSH Terms] OR "Perinatal Mortality"[MeSH Terms] OR "Placenta Diseases"[MeSH Terms:noexp] OR "diabetes, gestational"[MeSH Terms] OR "infant, newborn, diseases"[MeSH Terms:noexp] OR "Drug-Related Side Effects and Adverse Reactions"[MeSH Terms] OR "safety"[Title/Abstract] OR "adverse effect*"[Title/Abstract] OR "adverse event*"[Title/Abstract] OR "adverse outcome*"[Title/Abstract] OR "side effect*"[Title/Abstract] OR "toxicity"[Title/Abstract] OR "teratogen*"[Title/Abstract] OR "birth defect*"[Title/Abstract] OR "miscarriage"[Title/Abstract] OR "preterm"[Title/Abstract] OR "preeclampsia"[Title/Abstract] OR "pre eclampsia"[Title/Abstract] OR "Eclampsia"[Title/Abstract] OR "Stillbirth"[Title/Abstract] OR "neonatal morbidity"[Title/Abstract] OR "maternal morbidity"[Title/Abstract] OR "low birth weight"[Title/Abstract] OR "small for gestational age"[Title/Abstract] OR "congenital malformation*"[Title/Abstract] OR "congenital abnormalit*"[Title/Abstract] OR "pregnancy outcome*"[Title/Abstract] OR "pregnancy complication*"[Title/Abstract] OR "neonatal outcome*"[Title/Abstract] OR "maternal outcome*"[Title/Abstract] OR "birth outcome*"[Title/Abstract] OR "fetal outcome*"[Title/Abstract] OR "perinatal outcome*"[Title/Abstract] OR "gestational diabetes"[Title/Abstract] OR "Postpartum Hemorrhage"[Title/Abstract] OR "postpartum haemorrhage"[Title/Abstract] OR "NICU"[Title/Abstract] OR "neonatal intensive care"[Title/Abstract] OR "intrauterine growth restriction"[Title/Abstract] OR "IUGR"[Title/Abstract] OR "placental abruption"[Title/Abstract] OR "placenta previa"[Title/Abstract] OR "pregnancy loss"[Title/Abstract] OR "maternal safety"[Title/Abstract] OR "fetal safety"[Title/Abstract] OR "neonatal safety"[Title/Abstract]) AND ("Randomized Controlled Trial"[Publication Type] OR "Controlled Clinical Trial"[Publication Type] OR "Clinical Trial"[Publication Type] OR "Observational Study"[Publication Type] OR "Case Reports"[Publication Type] OR "Cohort Studies"[MeSH Terms] OR "Case-Control Studies"[MeSH Terms] OR "Longitudinal Studies"[MeSH Terms] OR "Follow-Up Studies"[MeSH Terms] OR "Pharmacovigilance"[MeSH Terms] OR "product surveillance, postmarketing"[MeSH Terms] OR "Adverse Drug Reaction Reporting Systems"[MeSH Terms] OR "Registries"[MeSH	**2280**

	Terms] OR "Randomized Controlled Trial"[Title/Abstract] OR "randomised controlled trial"[Title/Abstract] OR "quasi-experimental"[Title/Abstract] OR "quasi-randomized"[Title/Abstract] OR "quasi-randomised"[Title/Abstract] OR "non-randomized controlled"[Title/Abstract] OR "non-randomised controlled"[Title/Abstract] OR "cohort study"[Title/Abstract] OR "Cohort Studies"[Title/Abstract] OR "prospective study"[Title/Abstract] OR "prospective cohort"[Title/Abstract] OR "retrospective study"[Title/Abstract] OR "retrospective cohort"[Title/Abstract] OR "longitudinal study"[Title/Abstract] OR "follow-up study"[Title/Abstract] OR "case-control"[Title/Abstract] OR "case series"[Title/Abstract] OR "case report"[Title/Abstract] OR "Pharmacovigilance"[Title/Abstract] OR "post-marketing"[Title/Abstract] OR "postmarketing"[Title/Abstract] OR "spontaneous reporting"[Title/Abstract] OR "registry"[Title/Abstract] OR "register-based"[Title/Abstract]) AND humans[Filter] AND english[Filter] AND 2016/1/1:2026/6/1[pdat]	
**Scopus**<br>Date searched: 5 June 2026	**1** TITLE-ABS ("pregnan*" OR "antenatal" OR "prenatal" OR "gestation*" OR "maternal" OR "trimester*" OR "peripartum" OR "intrapartum" OR "pregnant women" OR "pregnant people")<br>**2** TITLE-ABS ("vitamin D" OR "cholecalciferol" OR "calcitriol" OR "ergocalciferol" OR "vitamin E" OR "tocopherol*" OR "omega-3" OR "omega 3" OR "zinc" OR "magnesium" OR "fish oil*" OR "docosahexaenoic" OR "eicosapentaenoic" OR "curcumin" OR "turmeric" OR "curcuma" OR "ginger" OR "Zingiber" OR "green tea" OR "EGCG" OR "epigallocatechin" OR "Boswellia" OR "frankincense" OR "boswellic acid*" OR "berberine" OR "resveratrol" OR "quercetin" OR "N-acetylcysteine" OR "N-acetyl cysteine" OR "acetylcysteine" OR "bromelain*" OR "alpha-lipoic acid" OR "alpha lipoic acid" OR "lipoic acid" OR "thioctic acid" OR "spirulina" OR "Arthrospira" OR "astaxanthin" OR "probiotic*" OR "coenzyme Q10" OR "CoQ10" OR "ubiquinone" OR "ubiquinol" OR "dietary supplement*")<br>**3** TITLE-ABS ("safety" OR "adverse effect*" OR "adverse event*" OR "adverse outcome*" OR "side effect*" OR "toxicity" OR "teratogen*" OR "birth defect*" OR "miscarriage" OR "preterm" OR "preeclampsia" OR "pre-eclampsia" OR "eclampsia" OR "stillbirth" OR "neonatal morbidity" OR "maternal morbidity" OR "low birth weight" OR "small for gestational age" OR "congenital malformation*" OR "congenital abnormalit*" OR "pregnancy outcome*" OR "pregnancy complication*" OR "neonatal outcome*" OR "maternal outcome*" OR "birth outcome*" OR "fetal outcome*" OR "perinatal outcome*" OR "gestational diabetes" OR "postpartum hemorrhage" OR "postpartum haemorrhage" OR "NICU" OR "neonatal intensive care" OR "intrauterine growth restriction" OR "IUGR" OR "placental abruption" OR "pregnancy loss" OR "maternal safety" OR "fetal safety" OR "neonatal safety") AND (DOCTYPE (ar) OR DOCTYPE (le) OR DOCTYPE (no))<br>**4** #1 and #2 and #3<br>**5** #4 and SUBJAREA (MEDI OR NURS OR PHAR)<br>**6** #5 AND NOT TITLE ("systematic review" OR "meta-analysis" OR "scoping review" OR "narrative review" OR "umbrella review" OR "literature review")<br>**7** #6 AND NOT TITLE-ABS ("iron" OR "ferrous" OR "ferric" OR "calcium" OR "folic acid" OR "folate" OR "vitamin B" OR "pyridoxine" OR "cobalamin" OR "riboflavin" OR "magnesium sulphate" OR "magnesium sulfate" OR "thiamine" OR "B12" OR "B6" OR "in vitro fertilization" OR "assisted reproduction" OR "mice" OR "mouse" OR "rat" OR "rats" OR "animal" OR "in vitro" OR "rabbit" OR "piglet")<br>**8** (LIMIT-TO (DOCTYPE (ar) OR DOCTYPE (le) OR DOCTYPE (no))<br>**9** LIMIT-TO (LANGUAGE,"English")<br>**10** PUBYEAR > 2015 AND PUBYEAR < 2027	**1975**
**Scopus,** sensitivity search, 22 October 2026	TITLE-ABS ("pregnan*" OR "antenatal" OR "prenatal" OR "gestation*" OR "maternal" OR "trimester*" OR "peripartum" OR "intrapartum" OR "pregnant women" OR "pregnant people") AND TITLE-ABS ("vitamin D" OR "cholecalciferol" OR "calcitriol" OR "ergocalciferol" OR "vitamin E" OR "tocopherol*" OR "omega-3" OR "omega 3" OR "zinc" OR "magnesium" OR "fish oil*" OR "docosahexaenoic" OR "eicosapentaenoic" OR "curcumin" OR "turmeric" OR "curcuma" OR "ginger" OR "Zingiber" OR "green tea" OR "EGCG" OR "epigallocatechin" OR "Boswellia" OR "frankincense" OR "boswellic acid*" OR "berberine" OR "resveratrol" OR "quercetin" OR "N-acetylcysteine" OR "N-acetyl cysteine" OR "acetylcysteine" OR "bromelain*" OR "alpha-lipoic acid" OR "alpha lipoic acid" OR "lipoic acid" OR "thioctic acid" OR "spirulina" OR "Arthrospira" OR "astaxanthin" OR "probiotic*" OR "coenzyme Q10" OR "CoQ10" OR "ubiquinone" OR "ubiquinol" OR "dietary supplement*" OR "herbal supplement" OR "nutraceutical") AND TITLE-ABS ("safety" OR "adverse effect*" OR "adverse event*" OR "adverse outcome*" OR "side effect*" OR "toxicity" OR "teratogen*" OR "birth defect*" OR "miscarriage" OR "preterm" OR "preeclampsia" OR "pre-eclampsia" OR "eclampsia" OR "stillbirth" OR "neonatal morbidity" OR "maternal morbidity" OR "low birth weight" OR "small for gestational age" OR "congenital malformation*" OR "congenital abnormalit*" OR "pregnancy outcome*" OR "pregnancy complication*" OR "neonatal outcome*" OR "maternal outcome*" OR "birth outcome*" OR "fetal outcome*" OR "perinatal outcome*" OR "gestational diabetes" OR "postpartum hemorrhage"	**3179**

	OR "postpartum haemorrhage" OR "NICU" OR "neonatal intensive care" OR "intrauterine growth restriction" OR "IUGR" OR "placental abruption" OR "pregnancy loss" OR "maternal safety" OR "fetal safety" OR "neonatal safety") AND NOT TITLE ("systematic review" OR "meta-analysis" OR "scoping review" OR "narrative review" OR "umbrella review" OR "literature review" OR "review") AND NOT TITLE-ABS ("mice" OR "mouse" OR "rat" OR "rats" OR "animal" OR "in vitro" OR "rabbit" OR "piglet") AND PUBYEAR > 2015 AND PUBYEAR < 2027 AND (LIMIT-TO (EXACTKEYWORD , "Human")) AND (LIMIT-TO (DOCTYPE , "ar") OR LIMIT-TO (DOCTYPE , "le") OR LIMIT-TO (DOCTYPE , "no")) AND (LIMIT-TO (LANGUAGE , "English"))	
**Web of Science**<br>Date searched: 5 June 2026	**1** TS=("pregnant" OR "antenatal" OR "prenatal" OR "maternal" OR "gestation" OR "trimester" OR "peripartum" OR "intrapartum" OR "pregnant women" OR "pregnant people")<br>**2** TS=("vitamin D" OR "cholecalciferol" OR "calcitriol" OR "ergocalciferol" OR "vitamin E" OR "tocopherol*" OR "omega-3" OR "omega 3" OR "fish oil*" OR "docosahexaenoic" OR "eicosapentaenoic" OR "magnesium" OR "zinc" OR "curcumin" OR "turmeric" OR "curcuma" OR "ginger" OR "Zingiber" OR "green tea" OR "EGCG" OR "epigallocatechin" OR "Boswellia" OR "frankincense" OR boswellic acid* OR "berberine" OR "resveratrol" OR "quercetin" OR "N-acetylcysteine" OR "N-acetyl cysteine" OR "acetylcysteine" OR bromelain* OR "alpha-lipoic acid" OR "lipoic acid" OR "thioctic acid" OR "spirulina" OR "Arthrospira" OR "astaxanthin" OR probiotic* OR "coenzyme Q10" OR "CoQ10" OR "ubiquinone" OR "ubiquinol" OR dietary supplement* OR herbal supplement* OR nutraceutical*)<br>**3** TS=("safety" OR "adverse effect*" OR "adverse event*" OR "adverse outcome*" OR "side effect*" OR "toxicity" OR teratogen* OR "birth defect*" OR "miscarriage" OR "preterm" OR "preeclampsia" OR "pre-eclampsia" OR "eclampsia" OR "stillbirth" OR "neonatal morbidity" OR "maternal morbidity" OR "low birth weight" OR "small for gestational age" OR congenital malformation* OR congenital abnormalit* OR "pregnancy outcome*" OR "pregnancy complication*" OR "neonatal outcome*" OR "maternal outcome*" OR "birth outcome*" OR "fetal outcome*" OR "perinatal outcome*" OR "gestational diabetes" OR "postpartum hemorrhage" OR "postpartum haemorrhage" OR "NICU" OR "neonatal intensive care" OR "intrauterine growth restriction" OR "IUGR" OR "placental abruption" OR "placenta previa" OR "pregnancy loss" OR "maternal safety" OR "fetal safety" OR "neonatal safety")<br>**4** TS=("iron" OR "ferrous" OR "ferric" OR "calcium" OR "folic acid" OR "folate" OR "vitamin B" OR "pyridoxine" OR "cobalamin" OR "riboflavin" OR "thiamine" OR "B12" OR "B6" OR "in vitro fertilization" OR "assisted reproduction" OR "magnesium sulphate" OR "magnesium sulfate" OR "rat" OR "rats" OR "mice" OR "rabbit" OR "piglet" OR "in vitro")<br>**5** TI=("systematic review" OR "meta-analysis" OR "scoping review" OR "narrative review" OR "umbrella review" OR "literature review")<br>**6** #1 and #2 and #3 not #4 not #5<br>**7** #6 and Article (Document Types) and English (Languages) and Nutrition Dietetics or Obstetrics Gynecology or Endocrinology Metabolism or Medicine General Internal or Pharmacology Pharmacy or Reproductive Biology or Toxicology or Developmental Biology or Food Science Technology (Web of Science Categories)<br>**8** search date between 01.01.2016 – 01.06.2026	**1452**
**Web of Science**<br>sensitivity search, 22 October 2026	#1 TS=("pregnant" OR "antenatal" OR "prenatal" OR "maternal" OR "gestation" OR "trimester" OR "peripartum" OR "intrapartum" OR "pregnant women" OR "pregnant people")<br>#2 TS=("vitamin D" OR "cholecalciferol" OR "calcitriol" OR "ergocalciferol" OR "vitamin E" OR "tocopherol*" OR "omega-3" OR "omega 3" OR "fish oil*" OR "docosahexaenoic" OR "eicosapentaenoic" OR "magnesium" OR "zinc" OR "curcumin" OR "turmeric" OR "curcuma" OR "ginger" OR "Zingiber" OR "green tea" OR "EGCG" OR "epigallocatechin" OR "Boswellia" OR "frankincense" OR boswellic acid* OR "berberine" OR "resveratrol" OR "quercetin" OR "N-acetylcysteine" OR "N-acetyl cysteine" OR "acetylcysteine" OR bromelain* OR "alpha-lipoic acid" OR "lipoic acid" OR "thioctic acid" OR "spirulina" OR "Arthrospira" OR "astaxanthin" OR probiotic* OR "coenzyme Q10" OR "CoQ10" OR "ubiquinone" OR "ubiquinol" OR dietary supplement* OR herbal supplement* OR nutraceutical*)<br>#3 TS=("safety" OR "adverse effect*" OR "adverse event*" OR "adverse outcome*" OR "side effect*" OR "toxicity" OR teratogen* OR "birth defect*" OR "miscarriage" OR "preterm" OR "preeclampsia" OR "pre-eclampsia" OR "eclampsia" OR "stillbirth" OR "neonatal morbidity" OR "maternal morbidity" OR "low birth weight" OR "small for gestational age" OR congenital malformation* OR congenital abnormalit* OR "pregnancy outcome*" OR "pregnancy complication*" OR "neonatal outcome*" OR "maternal outcome*" OR "birth outcome*" OR "fetal outcome*" OR "perinatal outcome*" OR "gestational diabetes" OR "postpartum hemorrhage" OR "postpartum haemorrhage" OR "NICU" OR "neonatal intensive care" OR "intrauterine growth restriction" OR "IUGR" OR "placental abruption" OR "placenta previa" OR "pregnancy loss" OR "maternal safety" OR "fetal safety" OR "neonatal safety")	**4087**

	#4 #1 AND #2 AND #3<br>#5 TS=("rat" OR "rats" OR "mouse" OR "mice" OR "rabbit" OR "piglet" OR "in vitro")<br>#6 #4 NOT #5<br>#7 TI=("systematic review" OR "meta-analysis" OR "scoping review" OR "narrative review" OR "umbrella review" OR "literature review" OR "review")<br>#8 #6 NOT #7 and Article (Document Types) and English (Languages)<br>Timespan 2016-01-01 to 2026-06-01 (publication date)	

Deduplication block

Step	Records
PubMed	2280
Scopus first search / sensitivity search	1975 / 3179 (1727 common)
Web of Science first search / sensitivity search	1452 / 4087 (1442 common)
Total records identified	12,973
Duplicates removed (within database, across databases, across search dates)	6811
Retracted publications removed	21
Records screened	6141 (3889 + 2252)
Records excluded at title/abstract	5977
Reports sought / not retrieved / assessed	164 / 8 / 156
Reports excluded at full text	60
Reports of included studies / unique studies	96 / 80 (16 companions, 12 study families)

Records were deduplicated in Zotero and Rayyan, MS Excel. New records were matched to the old ones by DOI, PMID and normalised title in the order PubMed, Scopus, Web of Science and only unmatched records were screened. 248 Scopus and 10 Web of Science from the first search were not retrieved by the sensitivity searches.

Table S3. Characteristics of included studies (*n*=80 unique studies, 96 publications)

No.	Author, Year Country	Design, Registry ID	N (enrolled) *n* analysed per group	**Population**	**Supplement**	**Dose**	**Trimester**	**Comparator**	Primary outcome (as stated by authors)	Maternal outcomes	**Fetal/neonatal outcomes**	Principal finding (as reported by authors)
1	Cooper et al. 2016 [94] United Kingdom	RCT - Secondary analysis ISRCTN82927713	1134 565 / 569	Healthy pregnant women	vitamin D	1000 IU/day	T2	Placebo	Primary: neonatal whole-body bone mineral content;	Postpartum haemorrhage;	Birth weight; Neonatal whole-body BMC; Birth length	Neonatal whole-body BMC not different (BMC 61.6 g (95% CI 60.3–62.8) vs 60.5 g (59.3–61.7), p=0.21)<br><br>Severe postpartum haemorrhage less frequent with cholecalciferol (severe PPH 17% placebo vs 12% cholecalciferol, p=0.01).
↳	Braithwaite et al. 2019 [90] United Kingdom	Secondary analysis RCT ISRCTN82927713	195 93/102	Healthy pregnant women	vitamin D	1000 IU/day	T2	Placebo	Secondary laboratory analysis of MAVIDOS	Inflammatory markers; Hepcidin; Ferritin	NR	Vitamin D did not affect hepcidin, iron status or CRP overall (hepcidin SD per SD 0.06 (95% CI −0.09 to 0.20), p=0.44)
↳	Moon et al. 2023 [103] United Kingdom	Secondary analysis RCT ISRCTN82927713	965 479 / 486	Healthy pregnant women	vitamin D	1000 IU/day	T2	Placebo	Post hoc analysis of MAVIDOS (primary: neonatal bone mass)	Preterm birth; Mode of delivery (c-section); Post-partum haemorrhage	Birth weight	No change in timing of birth or preterm birth. Spontaneous vaginal delivery (SVD) more likely with cholecalciferol (fewer instrumental deliveries).<br><br>SVD (versus instrumental or caesarean delivery) was more likely in women randomized to cholecalciferol RR 1.13 (95% CI 1.02–1.25); instrumental RR 0.68 (0.51–0.91); caesarean RR 0.94 (0.74–1.19).
↳	Citeroni-Clark et al. 2025 [93] United Kingdom	Secondary analysis RCT ISRCTN82927713	734 366 / 368	Healthy pregnant women	vitamin D	1000 IU/day	T2	Placebo	Secondary analysis of MAVIDOS	Preeclampsia/GH	NR	Vitamin D 1000 IU/day did not lower maternal BP or the incidence of PH or pre-eclampsia.
2	Yazdchi et al. 2016 [70] Iran	RCT - double blind, placebo controlled IRCT201306253140N11	72 36/ 36	Pregnant women with gestational diabetes mellitus diagnosed at 24-28 weeks of gestation	vitamin D	50 000 IU once, every 2 weeks	T2	Placebo	This study evaluated the effect of vitamin D supplementation on metabolic indices and hs-C-reactive protein (CRP) levels in GDM patients.	Inflammatory markers; Lipid profile; Glucose metabolism; GI symptoms; Serum 25-hydroxy vitamin D	NR	Vitamin D 50 000 IU/2 wk improved metabolic indices and hs-CRP in GDM.
3	Vaziri et al. 2016 [69] Iran	RCT IRCT201502031 0327N11	153 78 / 75	Nulliparous and multiparous	vitamin D	2000 IU/day	T3	Placebo	This clinical trial evaluated the effect of 2000 IU of vitamin D3 supplement per	Depressive symptoms (depression score)	NR	Vitamin D 2000 IU/day in late pregnancy reduced perinatal depression scores.

				pregnant women					day during late pregnancy on perinatal depression.			
4	Vaziri et al. 2016 [68] Iran	RCT IRCT20150 40910327N 13	134 67 / 67	Nulliparous and multiparous pregnant women	vitamin D	2000 IU/day	T3	Placebo	This clinical trial study, examined the effects of 2000 IU vitamin D3/day during late pregnancy on anthropometric measurements of infants in three separate time points and bone mass characteristics of mothers and their infants.	Maternal bone mass;	Birth weight; Infant anthropometric measurements at birth (infant weight, length and head circumference at birth, 4 and 8 weeks postnatal; infant bone mass);	No improvement in infant anthropometry or maternal/infant bone mass.
5	Zerofsky et al. 2016 [119] United States	RCT - double blind NCT014173 51	51 25 / 26	Healthy pregnant women.	vitamin D	400 IU/day or 2000 IU/day	T1	Active comparato r (400 IU/day vitamin D)	Primary: markers of adaptive immune function . The primary objective of the study was to compare the relative effects of 2 doses of vitamin D3 supplementation (2000 and 400 IU/d) on markers of adaptive immune function.	Inflammatory markers; Glucose metabolism; GI symptoms; Preeclampsia/GH;Mode of delivery (c-section);	Birth weight; Apgar	Vitamin D 2000 vs 400 IU/day increased 25(OH)D and regulatory T cells. No clinical outcome difference.<br><br>No AEs related to the supplementation.
6	Sasan et al. 2017 [65] Iran	RCT - double blind, placebo controlled IRCT20170 10131695N 1	142 70/ 72	Pregnant women with a history of preeclampsi a in previous pregnancies.	vitamin D	50 000 IU once every 2 weeks	T2	Placebo	The final outcome: recurrence of preeclampsia in the intervention group and control group.	Pre-eclampsia/GH; Mode of delivery; Abortion.	NR	Vitamin D 50 000 IU/2 wk reduced recurrent pre-eclampsia (PE).<br><br>PE risk in controls 1.94 × intervention (95% CI 1.02–3.71).<br><br>AEs/side effects: none of the subjects reported side effects.
7	Keshavarzi et al. 2017 [53] Iran	RCT - double blind, placebo controlled	192 128 (64/64) / 64	Pregnant women diagnosed with gestational diabetes mellitus with low serum 25-[OH] D levels (< 30 ng/mL);	vitamin D	Low-dose: 400 IU/day; High-dose: 50,000 IU (twice, at start and 21 days after)	T2	Placebo	Pregnancy outcome: neonatal anthropometric indices (birth weight, height, and head circumference), hypoglycemia, Apgar scores at minutes 1 and 5, and admission due to pathologic jaundice.	Mode of delivery (c-section)	Birth weight; Macrosomia; Apgar; Birth height; Head circumference; Neonatal hypoglycemia, jaundice	Low vs high dose vitamin D in GDM: no effect on pregnancy outcome except newborn height. Mean value of birth height was higher in the high-dose vitamin D group (50.13 ± 1.85 cm) vs. low-dose group (49.38 ± 2.18 cm; p= 0.02) and control group (49.02 ± 2.02 cm; p= 0.002). No statistically significant difference existed regarding mean height between low-dose group and control groups (p= 0.29)

8	Reynolds et al. 2017 [105] Ireland	Case report	1	A newborn girl born to Caucasian parents at 39 weeks' gestational age following an uncomplicated pregnancy.	vitamin D	4000 IU/day (1000 IU three times daily plus 1000 IU once daily)	Preconception/T1/T2/T3	Not applicable	Not applicable	Maternal total serum calcium	Congenital anomalies; Neonatal hypercalcaemia	Transient neonatal hypercalcaemia (3.09 mmol/L) after maternal 4000 IU/day from pre-conception to delivery. normalised within 2 weeks.
9	Ali et al. 2019 [35] Saudi Arabia	RCT - open label NCT03101150	164<br>81 - group 1 (400 UI) / 83 - group 2 (4000 UI)	Pregnant women with vitamin D3 deficiency (serum levels <25 nmol/L)	vitamin D	Group 1: 400 IU/day; Group 2: 4000 IU/day	T2	Active comparator: 400 UI/day vitamin D	Primary: pre-eclampsia	GDM; Preeclampsia/GH; Preterm birth; Mode of delivery (c-section);	Birth weight; IUGR	Fewer pre-eclampsia events with 4000 IU/day vs 400 IU/day (1.2% vs 8.6%; p<0.05), but RR 0.163 (95% CI 0.02–1.32, NNT=16) does not exclude no difference; IUGR lower with 4000 IU/day (9.6% vs 22.2%, p=0.027; RR 0.43, 0.19–0.94, NNT=79).
10	Motamed et al. 2019 [58] Iran	RCT - open label NCT03308487	73<br>36 /37	Healthy pregnant women	vitamin D	1,000 IU/day (group 1) and 2,000 IU/day (group 2)	T1	Active comparator: 1000 IU/day vitamin D	Primary: serum 25(OH)D;	Inflammatory markers; Premature delivery;	Birth weight; Apgar; Birth length; Head circumference	Vitamin D 2000 IU/day vs 1000 IU/day: higher 25(OH)D; significantly greater birth weight (3369.8±450.3 vs 3103.2±440.3 g, P=0.018), length (P=0.01) and head circumference (P=0.04). Apgar not different.
↳	Motamed et al. 2020 [59] Iran	Secondary analysis - RCT NCT03308487	73<br>36 / 37	Healthy pregnant women	vitamin D	1000 IU/day or 2000 IU/day	T1	Active comparator: 1000 IU/day vitamin D	Secondary analysis	Lipid profile; Glucose metabolism;	NR	Significant decrease in iPTH in both groups (−4.18 ± 7.5, p = 0.002 in 1000 IU/d group and −8.36 ± 14.17, p = 0.002 in 2000 IU/d group).
11	Corcoy et al. 2020 [107] United Kingdom, Ireland, Austria, Poland, Italy, Spain, Belgium (European multicenter)	RCT - double blind, placebo controlled ISRCTN70595832	154<br>79 / 75	Overweight/ obese pregnant women (BMI ≥29 kg/m2)	vitamin D	1600 IU/day	T2	Placebo	Primary: FPG, insulin resistance, GWG in late pregnancy	Glucose metabolism; GDM; Preeclampsia/GH; GWG; Mode of delivery (c-section); Adverse events	Apgar; Other perinatal outcomes	No major benefit for GDM prevention; small FPG reduction at 35–37 wk; FPG −0.14 mmol/L (95% CI −0.28 to 0.00) at 35–37 weeks. No AE related to vitamin D.

↳	Harreiter et al. 2022 [108] United Kingdom, Ireland, Austria, Poland, Italy, Spain, Belgium (European multicenter)	Secondary analysis RCT ISRCTN70595832	154 79/ 75	Pregnant women with overweight and obesity (BMI ≥29 kg/m2)	vitamin D	1600 IU/day	T2	Placebo	Secondary analysis of DALI; assess the effects of a vitamin D supplementation with 1600 IU Vitamin D3 per day starting in early pregnancy versus placebo on maternal and fetal lipid parameters, body fat distribution as well as pregnancy outcomes.	Lipid profile; GDM; Preeclampsia/GH; Preterm birth; Mode of delivery (c-section); Leptin levels	Birth weight; Neonatal sum of skinfolds; Birth height; Head and abdominal circumference	No effect on maternal or fetal lipid profile or body fat distribution.
12	Manasova et al. 2021 [102] Ukraine	RCT	54 29 / 25	Pregnant women with vitamin D insufficiency/deficiency and risk factors for preeclampsia	vitamin D	Group 1: 500 IU/day (as part of multivitamin-mineral complex); Group 2: 4000 IU/day until 16 weeks, then 2000 IU/day until end of pregnancy	T1	Active comparator (Multivitamin-mineral complex with 500 IU cholecalciferol only) - group 2	Prevention of preeclampsia by vitamin D administration	Preeclampsia/GH; Preterm birth; Mode of delivery (c-section); Blood loss in childbirth	Apgar; Fetal distress; CPAP therapy; IUGR	Vitamin D 500 IU/day (in multivitamin) vs 4000→2000 IU/day: more PE (OR 3.27 (95% CI 1.02–10.52), preterm birth (OR 2.74 (1.42–5.33) and caesarean (OR 2.37 ($p<0.05$)) in the low-dose group.
13	Xiaomang et al. 2021 [85] China	RCT - open label	450 135/ 134/ 138	Pregnant women with vitamin D deficiency	vitamin D	Low-dose: 400 IU/day; Medium-dose: 1500 IU/day; High-dose: 4000 IU/day	T1	Active comparator	Prevention of preeclampsia by vitamin D administration	GDM; Preeclampsia/GH; Preterm birth; Mode of delivery (c-section);	Birth weight; IUGR	Vitamin D3 in higher dose group reduced pre-eclampsia and the IUGR index, compared to low dose group.

14	Dahma et al. 2022 [95] Romania	Retrospective cohort	198<br>139 (63-2000 UI, 76-4000 UI) / 59 (no vitamin D)	Pregnant women at risk for preeclampsia, either with vitamin D supplementation (cases) or without vitamin D supplementation (controls) during pregnancy	vitamin D	2000 IU/day; 4000 IU/day	T1/T2/T3	No treatment	To evaluate the association between preeclampsia in pregnant women at risk, and their nutritional supplementation during pregnancy.	Preeclampsia/GH; Preterm birth; Mode of delivery (c-section)	Apgar; Congenital anomalies; NICU	No supplementation associated with higher frequence of pre-eclampsia; congenital anomalies 3.4%/3.2%/1.3% (p=0.691).
15	Ekmen et al. 2022 [42] Turkey	Retrospective cohort	60<br>28 / 32	Healthy pregnant women and their infants	vitamin D	1200 IU/day	T2	No treatment	To determine whether vitamin D administration throughout pregnancy could affect cord blood 25(OH)D levels, neonatal anthropometric measurements (height, head circumference, weight), and Apgar score.	NR	Birth weight; Apgar; Birth height; Head circumference; NICU admission	Higher cord 25(OH)D with 1200 IU/day; no difference in neonatal anthropometry, Apgar or NICU admission (Birth height p=0.806; head circumference p=0.838; NICU p=0.551).
16	Mirzaei-Azandaryani et al. 2022 [57] Iran	RCT - triple blind, placebo controlled IRCT20120718010324N59	84<br>43/ 41	Pregnant women at 8-10 weeks of gestation with vitamin D deficiency or insufficiency (less than 30 ng/ml) and BMI less than 30 kg/m2	vitamin D	4000 IU/day	T1	Placebo	Primary: FBG, FBI, HOMA-IR.	Glucose metabolism; GDM; depressive symptoms; musculoskeletal pain; miscarriage	NR	Vitamin D improved musculoskeletal pain; no effect on FBG, insulin, HOMA-IR, depression, GDM or abortion.
17	Cagiran et al. 2023 [41] Turkey	Non-randomized controlled trial	60<br>40 (20-400 IU/Day vitamin D, 20-600 IU/Day vitamin D) / 20	Pregnant women with vitamin D deficiency (serum vitamin D levels <20 ng/mL (groups 1 and 2) or sufficient vitamin D levels >20 ng/mL	vitamin D	400 IU/day (Group 1); 600 IU/day (Group 2)	T2	No treatment	Primary: GDM and gestational hypertension	GDM; Preeclampsia/GH; Preterm birth; Mode of delivery (c-section)	Birth weight	Vitamin D replacement did not prevent PIH or GDM nor change mode/timing of delivery

				(control group)								
18	Ku et al. 2024 [77] Singapore	RCT - open label NCT04841265	231 112 / 119	Pregnant women with overweight and obesity (BMI ≥25 kg/m2)	vitamin D	Intervention group: 800 IU/day (400 IU from standalone supplement + 400 IU from prenatal multivitamin); Control group: 400 IU/day vitamin D	T1	Active comparator: 400 IU/day vitamin D	Primary: maternal 25OHD and lipids;	Lipid profile; Glucose metabolism; GDM; Pre-eclampsia/GH; Mode of delivery (c-section); GWG; Preterm Birth;	Birth weight; Birth length; Head circumference; LBW	Vitamin D 800 vs 400 IU/day: higher 25OHD (25OHD adjusted difference 6.52 nmol/L (95% CI 2.74–10.30); lipid levels not different.
19	Yin et al. 2024 [86] China	RCT ChiCTR2100051914	1537 766 / 771	Pregnant women with serum 25(OH)D concentration < 75 nmol/L, including those with and without gestational diabetes mellitus or obesity	vitamin D	1600 IU/day in intervention group; 400 IU/day in control group	T2	Active comparator	Primary: gestational CVH markers	Inflammatory markers; Lipid profile; Glucose metabolism; Endothelial function markers (E-Selectin, ICAM) and SBP	NR	Vitamin D 1600 vs 400 IU/day improved gestational cardiovascular-health markers, especially in GDM/obesity.
20	Yu et al. 2025 [87] China	Retrospective cohort	98 49 / 49	Pregnant women with gestational diabetes mellitus	vitamin D	400 IU/day	T2/T3	No treatment	Primary: time to glycaemic control;.	Inflammatory markers; Lipid profile; Glucose metabolism; Preeclampsia/GH; Preterm birth; GWG; Postpartum hemorrhage; Malonyldialdehide	Fetal distress; Hypoglycemia, Asphyxia	Vitamin D 400 IU/day associated with faster glycaemic control and less insulin. Glycaemic control adjusted HR 2.30 (95% CI 1.50–3.52); insulin β −0.11 U/kg/day (−0.14 to −0.08).
21	Aminuddin et al. 2021 [72] Malaysia	RCT - Secondary analysis Secondary analysis of RCT after revised	299 151 / 148	Pregnant women, primigravidae	vitamin E	100 mg/day	T1/T2	Placebo	Primary: pre-eclampsia incidence;	Preeclampsia/GH; Preterm birth; Mode of delivery (c-section)	Birth weight; IUGR	Palm-oil vitamin E (TRF) associated with lower PIH and pre-eclampsia (secondary analysis, ISSHP reclassification). PIH adjusted OR 0.254 (95% CI 0.07–0.93); PE adjusted OR 0.030 (0.001–0.65)

		definision of preeclampsia according to ISSHP 2018										
22	Keenan et al. 2016 [114] United States	RCT - double blind, placebo controlled NCT01158976	64<br>43 / 21	Pregnant women from low income environments	omega-3	450 mg DHA per day; (90 mg EPA per day; 40 mg DPA per day)	T2	Placebo	Testing the effects of prenatal DHA supplementation on birth outcomes and infant development.	Gestational age	Birth weight; Apgar; infant cortisol response and Bayley scales at about 3 months of age	DHA 450 mg/day: infants of women living in urban, low-income environments who received prenatal DHA supplementation had more optimal birth outcomes including higher birth weight and higher 1-minute Apgar scores, and demonstrated more modulated cortisol response to a stressor at 3 months of age.
23	Faraji et al. 2016 [43] Iran	RCT - triple blind, placebo controlled IRCT2013100914957N1	92<br>45 / 47	Healthy pregnant women with first to fifth parity	omega-3	1000 mg/day fish oil containing 180 mg EPA and 120 mg DHA	T2	Placebo	To determine the impact of supplementation with fish oil during the second half of gestation on lipid profiles of pregnant women.	Lipid profile; Mode of delivery	NR	Fish oil 1000 mg/day changed lipid profile vs placebo (TC within-group MD 26.82 (95% CI 11.44–42.19); TG 69.49 (49.48–89.49))
↳	Ostadrahimi et al. 2017 [61] Iran	RCT - triple blind, placebo controlled IRCT2013100914957N1	150<br>75 / 75	Pregnant women with first to fifth pregnancy	omega-3	1000 mg/day fish oil containing 180 mg EPA and 120 mg DHA	T2	Placebo	Primary: birth weight;	GI symptoms; Preeclampsia/GH; Preterm birth; Maternal serum DHA fatty acid	Birth weight; Congenital anomalies; Birth length; Head circumference	Birth weight higher but not significant; LBW frequency significantly lower; preterm not different; Minor GI side effects not different between groups.
24	Kuper et al. 2017 [115] United States	RCT - Secondary analysis N160429005	851<br>434 / 417	Pregnant women with a singleton pregnancy and a history of a previous singleton spontaneous preterm delivery. 6% were smokers (136 women) and 84% were nonsmokers (715 women).	omega-3	1200 mg eicosapentaenoic acid (EPA) and 800 mg docosahexaenoic acid (DHA) for a total of 2000 mg daily	T2	Placebo	Primary: spontaneous preterm delivery (secondary analysis by smoking status)	Preeclampsia/GH; Preterm birth	Birth weight; SGA; Neonatal composite outcome (retinopathy of prematurity, intraventricular hemorrhage grade III or IV, patent ductus arteriosus, necrotizing enterocolitis, sepsis, respiratory morbidity, or perinatal death), NICU admission	In smokers, omega-3 reduced spontaneous preterm delivery and LBW; no effect in non-smokers (secondary analysis). Smokers: preterm RR 0.56 (95% CI 0.36–0.87); LBW RR 0.57 (0.36–0.90); non-smokers RR 1.04 (0.84–1.29).

25	Jamilian et al. 2018 [50] Iran	RCT - double blind, placebo controlled IRCT201610015623N90	40<br>20 / 20	Pregnant women with gestational diabetes mellitus	omega-3	1000 mg/day fish oil (180 mg EPA and 120 mg DHA per capsule)	T2	Placebo	Primary: PPAR-γ gene expression	Inflammatory markers; Lipid profile; Glucose metabolism; Gestational age at delivery; Polyhydramnios,	Birth weight; Apgar; Birth length; Head circumference;	Fish oil downregulated IL-1 and TNF-α expression, reduced hs-CRP and FPG.
26	Alkhafajy et al. 2018 [34] Iraq	Non-randomised controlled interventional study	280<br>140 / 140	Singleton pregnant women at high risk of preterm labour	omega-3	1000 mg/day	T2	No treatment	Primary aim: delivery before/after 37 weeks	GI symptoms; Preterm birth	Birth weight; NICU admissions.	Omega-3 prolonged pregnancy, increased birth weight, reduced neonatal admissions; Tolerable GI upset was the only adverse effect. Preterm 18.5% vs 32.8%, p<0.05; NICU admissions 22.8% vs 46.4%, p<0.05.
27	Makrides et al. 2019 [123] Australia	RCT - double blind, placebo controlled ACTRN12613001142729	5474<br>2731 women/ 2734 pregnancies / 2743 women/ 2752 pregnancies	Pregnant women with single or multiple fetuses	omega-3	900 mg omega-3 PUFA daily (approximately 800 mg DHA and 100 mg EPA per day)	T1	Placebo	Primary: early preterm delivery <34 weeks;	GI symptoms; GDM; Preeclampsia/GH; Preterm birth; Mode of delivery (c-section); Serious adverse events:: misscariage	Birth weight; LGA; Serious adverse events (perinatal/neonatal death, major congenital abnormality)	No reduction in early preterm delivery; higher rate of very LGA infants; minor GI disturbances more common with n-3; SAEs percentages did not differ.<br><br>Early preterm: 2.2% vs 2.0%, adjusted RR 1.13 (95% CI 0.79–1.63), P=0.50; very LGA adjusted RR 1.30 (1.02–1.65); any maternal SAE 14 (0.5%) vs 10 (0.4%); any infant/pregnancy SAE 142 (5.0%) vs 112 (4.0%), RR 1.27 (0.99–1.62).
↳	Simmonds et al. 2020 [124] Australia	Secondary analysis RCT ACTRN12613001142729	5070<br>2523 women/ 2525 pregnancies / 2536 women/ 2545 pregnancies	Women with singleton pregnancies	omega-3	900 mg omega-3 PUFA daily (approximately 800 mg DHA and 100 mg EPA per day)	T1	Placebo	Exploratory analysis of the ORIP trial (early preterm birth)	Preterm birth	NR	Exploratory: benefit confined to women with low baseline omega-3 status, harm signal with high baseline status.<br><br>Baseline status ≤4.1%: 0.73% vs 3.16%, RR 0.23 (95% CI 0.07–0.79); >4.9%: 2.20% vs 0.97%, RR 2.27 (1.13–4.58)

28	Olsen et al. 2019 [79] China	RCT - triple blind, placebo controlled NCT02770456	5531 1825 high fish oil group; 1851 low fish oil group / 1855	Pregnant women with low and high fish intake	omega-3	2.0 g/day (high fish oil group); 0.5 g/day (low fish oil group); 0 g/day (control group), divided in 4 capsules daily	T2	Placebo	Primary: hazard of spontaneous delivery in the preterm period	Preterm birth	NR	Fish oil did not reduce preterm delivery hazard in Chinese women (HRR <259 d: 1.04 (95% CI 0.70–1.53) low dose; 0.90 (0.60–1.35) high dose).
29	Gholami et al. 2020 [44] Iran	RCT IRCT2015072123269N1	339 160/ 179	Pregnant women with singleton pregnancy	omega-3	1000 mg fish oil per day containing 120 mg DHA and 180 mg EPA	T2	No treatment	Primary: incidence of preeclampsia, eclampsia, preterm labor, gestational age at delivery, birth weight and height, and the occurrence of gestational diabetes	GDM; Preeclampsia/GH; Preterm birth; Gestational age at labor;	Birth weight; IUGR; Height and head circumference	Fish oil supplementation from 20 weeks of gestation significantly increased gestational age at delivery by approximately 4 days in pregnant women aged 18-35 years. However, it failed to decrease the incidence of preterm birth, preeclampsia, eclampsia, intrauterine growth restriction, or gestational diabetes. The supplement did not significantly affect birth weight, height, or head circumference.
30	Khandelwal et al. 2021 [127] India	RCT - double blind, placebo controlled NCT01580345	957 478 / 479	Healthy pregnant women with singleton pregnancy	omega-3	400 mg/day DHA (2x200 mg/capsule per day)	T2	Placebo	Primary: newborn anthropometry;	Preterm birth	Birth weight; Apgar; Birth length; LBW; Head circumference	DHA 400 mg/day did not change newborn anthropometry.
31	Monthé-Drèze et al. 2021 [116] United States	RCT - double blind, placebo controlled NCT00957476	48 24 / 24	Pregnant women with overweight and obesity (BMI ≥25 kg/m2)	omega-3	2000 mg/day (800 mg DHA plus 1200 mg EPA-devided in 4 capsules)	T1	Placebo	Co-primary (parent pilot): maternal insulin sensitivity and inflammation	Inflammatory markers; Glucose metabolism; Preterm birth; Placental lipid storage	Birth weight; neonatal fat-free mass	Pilot: n-3 associated with higher neonatal fat-free mass and longer gestation. (β: 218 g; 95% CI 49, 387) but not with % body fat or fat mass. Birthweight for gestational age z-score (−0.17 ± 0.67 vs. −0.61 ± 0.61 SD unit, p = 0.02) was higher, and gestation longer (40 (38.5, 40.1) vs. 39 (38, 39.4) weeks, p = 0.02), in the treatment vs. placebo group

32	Garmendia et al. 2021 [113] Chile	RCT - triple blind NCT02574767	1002<br>250 /252/ 249/251	Obese and overweight pregnant women with medium-low income	omega-3	800 mg/day (Groups 1 and 2) or 200 mg/day (Groups 3 and 4) of docosahexaenoic acid, fish oil EPA-DHA	T1	Active comparator (routine counseling + 200 mg/day DHA)	Primary: GDM (mothers); macrosomia and insulin resistance (neonates)	Glucose metabolism; GI symptoms; GDM; Preeclampsia/PH; Preterm birth; Mode of delivery (c-section); Maternal hospitalization; SAEs	Birth weight; Congenital anomalies; Macrosomia; Neonatal insulin resistance; Hyperbilirubinemia	No reduction in GDM, macrosomia or neonatal insulin resistance; 238 SAEs recorded, none judged related to DHA; 479 non-serious events possibly related - nausea, heartburn, flatulence, reflux.<br><br>SAEs: abortion 2.5% (25), neonatal/fetal death 1.5% (15), maternal hospitalisation 13.7% (137), congenital malformation 2.7% (27), P=0.185; cord glucose lower in Group 2 vs 4, β −0.11 (95% CI −0.20 to −0.01)
33	Carlson et al. 2021 [109] United States	RCT - double blind NCT02626299	1032<br>492/ 540	Pregnant women with singleton pregnancies	omega-3	1000 mg/day (intervention: 800 mg algal oil + 200 mg prenatal supplement); 200 mg/day (control group: 200 mg prenatal supplement) DHA	T1/T2	Active comparator: 200 mg DHA/day	Primary: early preterm birth <34 weeks by dose and enrolment DHA status;	GDM; Preeclampsia/GH; Preterm birth; Mode of delivery (c-section); Maternal RBC DHA% at delivery; Adverse events and SAEs (chorioamnionitis, PROM, pyelonephritis)	Birth weight; Birth length; Head circumference; NICU admission; Neonatal adverse events: feeding, genitourinary, neurologic, musculoskeletal	1000 mg/day DHA likely superior to 200 mg/day for early preterm birth; fewer maternal and neonatal SAEs with the higher dose (Bayesian posterior probabilities).<br><br>EPB 1.7% (9/540) vs 2.4% (12/492), pp 0.91 for superiority; fewer SAEs with higher dose, pp>0.90;
↳	Valentine et al. 2021 [118] United States	Secondary analysis RCT NCT02626299	902<br>465 / 437	Pregnant women with singleton pregnancies	omega-3	1000 mg/day (intervention: 800 mg algal oil + 200 mg prenatal supplement); 200 mg/day (control group: 200 mg prenatal supplement) DHA	T2	Active comparator: 200 mg DHA/day	Primary (this analysis): gestational age and preterm birth <37 wk	Inflammatory markers; Preeclampsia/GH; Preterm birth; sRAGE (soluble Receptor for Advanced Glycation End Products)	NR	Higher-dose DHA modulated sRAGE/IL-6; associated with lower preterm birth.

34	Mu et al. 2024 [78] China	Retrospective cohort	379 105 / 274	Pregnant women with a history of pregnancy loss	omega-3	465 mg EPA and 375 mg DHA per capsule	T1/T2	No treatment	To explored the effectiveness of omega-3 fatty acids in regulating lipid levels during pregnancy.	Lipid profile	NR	Omega-3 reduced triglycerides during pregnancy; no effect on TC, LDL-C, HDL-C. TG adjusted β −0.300 (95% CI −0.445 to −0.154), p<0.001.
35	Zielinsky et al. 2024 [120] Brazil	RCT - double blind, placebo controlled NCT02565290	48 24 / 24	Pregnant women without cardiac or extracardiac fetal anomalies, singleton pregnancies	omega-3	450 mg/day DHA	T3	Placebo	Primary: maternal PGE2 and ductal pulsatility index	Maternal serum PGE2 levels	Fetal ductus arteriosus pulsatility index; Doppler echocardiographic parameters	DHA 450 mg/day in T3 did not inhibit PGE2 or constrict the ductus arteriosus.
36	Phattharachindanuwong et al. 2025 [80] Thailand	RCT NCT06302023	61 30 / 31	Pregnant women diagnosed with threatened preterm labor at 24 to 34 weeks of gestation	omega-3	1000 mg/day fish oil EPA-DHA	T2	No treatment	Primary: preterm birth rate; side effects of the DHA supplement and its impact on newborns	GI symptoms; Preterm birth; Mode of delivery (c-section); Peripartum infections; Postpartum hemorrhage	Birth weight; NICU admissions	DHA after threatened preterm labour did not reduce preterm birth (23.3% (7/30) vs 25.8% (8/31), p=0.82); no side effects reported.
37	Wang et al. 2025 [84] China	Retrospective cohort	278 60 / 218	Pregnant women with previous pregnancy loss and infertility history	omega-3	840 mg/day (465 mg EPA + 375 mg DHA per capsule)	T1/T2/T3	No treatment	To compare blood lipids between the medicated and non-medicated groups during the same pregnancy period, to exclude errors arising from elevated blood lipids due to the increase in gestational weeks, and to better compare the changes in blood lipids after Omega-3 fatty acid interventions, as well as to evaluate the effects on pregnancy outcomes and neonatal outcomes by following up on pregnancy outcomes in all participants.	Lipid profile; Preeclampsia/GH; Preterm birth; Liver and kidney function, coagulation function	Birth weight; SGA; LGA; Macrosomia	No difference in live birth, pregnancy loss, gestational weeks or birth weight.
38	Carvajal et al. 2026 [111] Chile	RCT - double blind, placebo controlled NCT02336243	809 404 / 405	Singleton pregnant women from low- and middle-income populations	omega-3	200 mg each x3/day; 600 mg/day algal derived DHA	T1	Placebo	Primary: preterm birth rate, severe fetal growth restriction, preeclampsia;	GI symptoms; Preeclampsia/GH; Preterm birth; Serious adverse events	Birth weight; SGA; Apgar; NICU admissions; Birth length; Head circumference	No effect on composite of preterm birth ≤34 wk, early-onset PE, severe FGR; no SAEs recorded; minor AEs 4.5% vs 4%.<br><br>Composite 3.47% vs 2.96%, RR 1.17 (95% CI 0.55–2.50), P=0.685; severe FGR RR 0.79 (0.22–2.95); early PE RR 0.50 (0.09–2.73).

39	Karamali et al. 2016 [51] Iran	RCT - double blind, placebo controlled IRCT201601035623N63	60 30/ 30	Pregnant primigravida women diagnosed with gestational diabetes mellitus	probiotic	$2x10^9$ CFU/g, each of *Lactobacillus acidophilus; L. casei; Bifidobacterium bifidim,* once daily	T2	Placebo	Primary: glucose homeostasis	Lipid profile; Glucose metabolism; Mode of delivery (c-section)	Birth weight; Newborn height; Macrosomia.	Probiotics improved glycaemic control (FPG(-9.2 ± 9.2 mg/dL vs +1.1 ± 12.2 mg/dL, P < 0.001), serum insulin levels (-0.8 ± 3.1 IU/mL vs +4.5 ± 10.6 IU/mL, P = 0.01), HOMA-IR (-0.4 ± 0.9 vs +1.1 ± 2.5, P = 0.003)), TG (-1.6 ± 59.4 mg/dL vs +27.1 ± 37.9 mg/dL, P = 0.03) and VLDL (-0.3 ± 11.9 mg/dL vs +5.4 ± 7.6 mg/dL, P = 0.03).
40	Di Pierro et al. 2016 [96] Italy	Non-randomised controlled, open label study	406 127 / 279	Healthy pregnant women	probiotic	*Enterococcus faecium L3* $5×10^9$ CFU/dose; *Bifidobacterium animalis ssp. lactis BB12* $3×10^9$ CFU/dose; *Lactobacillus casei RO215* $3×10^9$ CFU/dose; *Lactococcus lactis ssp. lactis SP 38* 3×10*9* CFU/dose	T3	No treatment	To examine whether administration of a probiotic mixture containing Enterococcus faecium L3 during the last 10 weeks of pregnancy could affect outcomes such as occurrence of Streptococcus agalactiae colonization and premature rupture of membranes.	GI symptoms; PROM (premature rupture of membranes); Mode of delivery (c-section)	NR	Probiotic mixture associated with fewer GI symptoms and PROM.
41	Jafarnejad et al. 2016 [47] Iran	RCT - double blind, placebo controlled	72 37 / 35	Pregnant women with gestational diabetes mellitus	probiotic	$112.5 × 10^9$ CFU/capsule VSL#3	T2/T3	Placebo	Primary: change in fasting glucose	Inflammatory markers; Glucose metabolism	NR	VSL#3 improved insulin resistance indices; no change in FPG or HbA1c.

42	Wickens et al. 2017 [125] New Zealand	RCT - double blind, placebo controlled ACTRN12612000196842	373 184/ 189	Pregnant women with personal or partner history of atopic disease	probiotic	>6×10⁹ CFU daily (*Lactobacillus rhamnosus* HN001)	T1	Placebo	Primary outcome: investigating the effects of the probiotic HN001 on the prevalence of GDM.	Glucose metabolism; GDM; Preterm birth; GWG; Mode of delivery (c-section)	Birth weight; Apgar; Infant length, ponderal index, head circumference; NICU	GDM prevalence lower with HN001 by the NZ definition (2.1% vs 6.5%, P=0.03) but not by IADPSG (P=0.08); IADPSG: RR 0.59 (95% CI 0.32–1.08); NZ definition: 2.1% (0.6–5.2) vs 6.5% (3.5–10.9); women ≥35 y RR 0.31 (0.12–0.81)
↳	Chen et al. 2021 [122] New Zealand	Secondary analysis RCT ACTRN12612000196842	348 172/ 176	Pregnant women with personal or partner history of atopic disease	probiotic	>6×10⁹ CFU daily (*Lactobacillus rhamnosus* HN001)	T1	Placebo	Secondary analysis	Lipid profile; Glucose metabolism; GDM; Lower fasting conjugated bile acids	NR	HN001 lowered conjugated bile acids and fasting glucose
43	Hajifaraji et al. 2017 [45] Iran	RCT - double blind, placebo controlled IRCT201405181597N3	56 29/ 27	Nulliparous pregnant females with gestational diabetes mellitus diagnosed at 24-28 weeks of gestation	probiotic	180 mg/day (>4×10⁹ CFU per capsule *Lactobacillus acidophilus LA-5, Bifidobacterium BB-12, Streptococcus thermophilus STY-31 and Lactobacillus delbrueckii bulgaricus LBY-27*)	T2	Placebo	To investigate the effect of probiotic supplements on systolic and diastolic blood pressure in pregnant females diagnosed with GDM	Preeclampsia/GH	NR	Probiotics prevented SBP rise and lowered DBP in GDM.
↳	Hajifaraji et al. 2018 [46] Iran	RCT - double blind, placebo controlled IRCT201405181597N3	56 29 / 27	Nulliparous pregnant females with gestational diabetes mellitus diagnosed at 24-28 weeks of gestation	probiotic	180 mg/day (4x10⁹ CFU per capsule *Lactobacillus acidophilus LA-*	T2	Placebo	To measure the effect of a probiotic supplement capsule containing four bacterial strains in comparison with a placebo on inflammatory and oxidative stress biomarkers among	Inflammatory markers; Malondialdehyde levels; Glutathione reductase; Erythrocyte glutathione peroxidase	NR	TNF-α serum levels decreased in the intervention group and increased in the placebo group. Intergroup comparison indicated that the serum TNF-α levels were significantly lower in probiotic group as compared to placebo (-0.04±0.10 vs 0.38±0.12; p=0.009). The serum IL-6 levels had decreased in both groups and there was not a significant difference

						5, Bifidobacterium BB-12, Streptococcus thermophilus STY-31 and Lactobacillus delbrueckii bulgaricus LBY-27)			women with newly-diagnosed GDM			between two groups. Hs-CRP decreased in probiotic group an increase in placebo group at the after-treatment evaluations, adding probiotics to the routine diet results in significantly lower mean hs-CRP values (p=0.019).
44	Kirihara et al. 2018 [76] Japan	Retrospective cohort	121 45 / 76	Pregnant women at high risk of preterm birth.	probiotic	3-6 g/day containing Streptococcus faecalis 10 mg/g, Clostridium butyricum 50 mg/g and Bacillus mesentericus 50 mg/g	T1	No treatment	To evaluate the effect of probiotics on perinatal outcomes including preterm birth, onset of chorioamnionitis (CAM) or funisitis and vaginal flora in pregnant women at high risk of preterm birth.	Preterm birth; Chorioamnionitis; Funisitis;	Birth weight;	Probiotics associated with lower preterm birth <32 wk and chorioamnionitis. Preterm <32 wk adjusted OR 0.05 (95% CI 0.01–0.71)
45	Babadi et al. 2019 [39] Iran	RCT - double blind, placebo controlled IRCT20171010036697N1	48 24 / 24	Primigravida women with gestational diabetes mellitus not requiring oral hypoglycemic agents at enrollment	probiotic	2×10^9 CFU/g each of *Lactobacillus acidophilus, Lactobacillus casei, Bifidobacterium bifidum, and Lactobacillus fermentum,*	T2	Placebo	Primary: PPAR-γ gene expression	Inflammatory markers; Lipid profile; Glucose metabolism; Malondialdehyde; Nitric oxide, Total antioxidant capacity	NR	Probiotics upregulated PPAR-γ and reduced FPG, insulin, HOMA-IR<br><br>FPG β −3.43 mg/dL (95% CI −6.48 to −0.38); insulin β −2.29 (−3.60 to −0.99)

						once daily						
46	Callaway et al. 2019 [121] Australia	RCT - double blind, placebo controlled ACTRN12611001208998	411 207 / 204	Overweight and obese pregnant women (BMI ≥25 kg/m²)	probiotic	>1×10⁹ colony-forming units each per day (*Lactobacillus rhamnosus* and *Bifidobacterium animalis* subspecies lactis)	T2	Placebo	Primary: GDM at 28 weeks (IADPSG)	Glucose metabolism; GDM; Preeclampsia/GH; Preterm birth; GWG	SGA; Congenital anomalies; Neonatal special care, jaundice, hypoglycemia, birth injury, infant body composition	Probiotics did not prevent GDM; fasting glucose slightly higher with probiotics; no difference in congenital anomalies<br><br>GDM 18.4% vs 12.3%, RR 1.62 (95% CI 0.91–2.89), P=0.10; fasting glucose MD 1.53 mg/dL (0.0095–3.06)
47	Halkjær et al. 2020 [97] Denmark	RCT - double blind, placebo controlled NCT02508844	50 25 / 25	Nulliparous obese pregnant women (BMI 30-35 kg/m²)	probiotic	450 billion CFU/day	T2	Placebo	Primary: GWG and glucose homeostasis (feasibility)	Glucose metabolism; GDM; Preeclampsia/GH; GWG; Mode of delivery (c-section)	Birth weight; LGA	Feasible; modulated microbiota; no effect on GWG, GDM, HbA1c or birth weight
48	Browne et al. 2021 [91] The Netherlands	Randomised pilot trial– double blind, placebo controlled NTR6219	40 20 / 20	Healthy pregnant women with elevated depressive symptoms and/or anxiety	probiotic	2000 mg/day (2.5 × 10^9 CFU/g; 2 g daily dosage containing *Bifidobacterium bifidum W23, Bifidobacterium lactis W51, Bifidobacterium lactis*	T2	Placebo	Primary: feasibility and acceptability;	GI symptoms; Depressive symptoms; Side effects and serious adverse events.	NR	Pilot feasible; no difference in mood outcomes; side effects and SAEs not different

						W52, Lactobacillus acidophilus W37, Lactobacillus brevis W63, Lactobacillus casei W56, Lactobacillus salivarius W24, Lactococcus lactis W19 and Lactococcus lactis W58)						
49	Shahriari et al. 2021 [67] Iran	RCT - double blind, placebo controlled IRCT2016110111020N6	507 241/ 266	High-risk pregnant women	probiotic	500 mg/day *Lactobacillus acidophilus LA1* (>7.5 × 10^9 CFU), *Bifidobacterium longum sp54 cs* (>1.5 × 10^9 CFU), and *Bifidobacterium bifidum sp9 cs* (>6 × 10^9 CFU)	T2	Placebo	Primary: GDM risk	Glucose metabolism; GDM; Preeclampsia/GH; Mode of delivery (c-section)	Birth weight; IUGR; Macrosomia	Probiotics did not reduce GDM in high-risk women (GDM 41.9% vs 40.2%, P=0.78)

50	Cardoso et al. 2016 [126] India	Non-randomised controlled study	200 100 / 100	Healthy pregnant women	combination	Vitamin C 500 mg/day; Vitamin E 400 IU/day	T2	No treatment	Primary: incidence of preeclampsia	Preeclampsia/GH; Preterm birth; Mode of delivery (c-section)	Birth weight; Apgar; Neonatal deaths	Vitamin C+E group had lower pre-eclampsia, severe pre-eclampsia, preterm delivery and neonatal admissions. PE RR 0.54 (95% CI 0.33–0.87); severe PE RR 0.28 (0.12–0.63)
51	Jamilian et al. 2017 [48] Iran	RCT IRCT201507035623N47	60 30 / 30	Pregnant women with gestational diabetes mellitus	combination	1000 mg omega-3 fatty acids from flaxseed oil (containing 400 mg alpha-linolenic acid) plus 400 IU vitamin E per day	T2	Placebo	Primary: MDA	Inflammatory markers; Mode of delivery (c-section); TAC	SGA; Apgar; Hyperbilirubinemia	Omega-3 (flaxseed) + vitamin E improved TAC, MDA, NO and reduced newborn hyperbilirubinaemia
52	Jamilian et al. 2017 [49] Iran	RCT - double blind, placebo controlled IRCT201605135623N78	140 35/35/35/35	Pregnant women with gestational diabetes mellitus	combination	Vitamin D: 50,000 IU every 2 weeks; Omega-3: 1000 mg/day containing 360 mg EPA and 240 mg DHA	T2	Placebo	Primary: markers of insulin metabolism	Lipid profile; Glucose metabolism	NR	Vitamin D + omega-3 improved FPG, insulin, HOMA-IR, QUICKI and lipids vs each alone and placebo;<br><br>FPG change −7.3±7.8 vs −6.9±6.6 vs −4.0±2.5 vs −1.0±11.4 mg/dL, P<0.001
53	Razavi et al. 2017 [62] Iran	RCT - double blind, placebo controlled RCT201701305623N106	120/ 30/30/30/30	Pregnant women with gestational diabetes mellitus	combination	1000 mg omega-3 fatty acids containing 180 mg EPA and 120 mg DHA twice a day +	T2	Placebo	Primary: inflammatory markers	Inflammatory markers; Preeclampsia/GH; Preterm birth; Mode of delivery (c-section); TAC; Glutathione; Malondialdehyde; Polyhydramnios	Birth weight; Apgar; Macrosomia; Birth length; Head circumference	Vitamin D + omega-3 improved hs-CRP, TAC, MDA; no difference in PE (p=0.96), preterm (p=0.39), birth weight (p=0.48)

						vitamin D placebo (n = 30); or 50,000 IU vitamin D every 2 weeks + omega-3 fatty acids placebo (n = 30); or 50,000 IU vitamin D every 2 weeks + 1000 mg omega-3 fatty acids twice a day (n = 30)						
54	Karamali et al. 2018 [52] Iran	RCT - double blind, placebo controlled IRCT201704205623N108	60 30 / 30	Pregnant women with gestational diabetes mellitus	combination	2×10^9 CFU/g each of *Lactobacillus acidophilus strain T16; L. casei strain T2; Bifidobacterium bifidum strain T1* and inulin 800 mg, once daily	T2/T3	Placebo	Primary: inflammatory markers	Inflammatory markers; Preeclampsia/GH; Mode of delivery (c-section); Plasma malondialdehyde; Total antioxidant capacity; Total glutathione.	Birth weight; Apgar; Macrosomia; Birth Length; Hyperbilirubinemia	Synbiotic reduced hs-CRP and caesarean rate; improved oxidative markers

55	Maktabi et al. 2018 [54] Iran	RCT - double blind, placebo controlled IRCT20170513033941N24	60 30/ 30	Pregnant women with gestational diabetes mellitus	combination	250 mg/day magnesium oxide and 400 IU/day vitamin E	T2	Placebo	Primary: glycemic control	Lipid profile; Glucose metabolism	NR	Mg + vitamin E improved glycaemic control and lipids except HDL-C.<br><br>FPG β −5.20 mg/dL (95% CI −7.88 to −2.52); TG β −50.31 (−67.58 to −33.04).
56	Pellonperä et al. 2019 [104] Finland	RCT - double blind, placebo controlled NCT01922791	438 109/110/109/110	Overweight and obese pregnant women with prepregnancy BMI ≥25 kg/m²	combination	Fish oil: 2.4 g of n-3 fatty acids; 1.9 g docosahexaenoic acid and 0.22 g eicosapentaenoic acid daily; Probiotics: 1x10^10 CFU each of *Lactobacillus rhamnosus* HN001 and *Bifidobacterium animalis ssp. lactis* 420, daily	T1	Placebo	Primary: GDM incidence and change in FPG	Glucose metabolism; GI symptoms; GDM; Preeclampsia/GH; Preterm birth; Mode of delivery (c-section); Postpartum hemorrhage	Birth weight; SGA; Macrosomia; Congenital anomalies;	Fish oil and/or probiotics did not lower GDM incidence;<br><br>AEs of capsules 28%, no between-group difference;
↳	Houttu et al. 2020 [98] Finland	Secondary analysis - RCT NCT01922791	438 109/110/109/110	Overweight and obese pregnant women with prepregnancy BMI ≥25 kg/m²	combination	Fish oil: 2.4 g of n-3 fatty acids; 1.9 g docosahexaenoic acid (DHA) and 0.22 g eicosapentaenoic acid	T1	Placebo	Secondary analysis	Inflammatory markers; Glucose metabolism; GDM; MMP-8 and IGFBP-1.	NR	No intervention effect on hsCRP, IGFBP-1, MMP-8;

						(EPA) per day; Probiotics 1x10^10 CFU each of *Lactobacillus rhamnosus* HN001 and *Bifidobacterium animalis ssp. lactis* 420, daily						
↳	Houttu et al. 2024 [99] Finland	Secondary analysis - RCT NCT01922791	438 109 /110 /109 /110	Overweight and obese pregnant women with prepregnancy BMI ≥25 kg/m²	combination	Fish oil: 2.4 g of n-3 fatty acids; 1.9 g docosahexaenoic acid (DHA) and 0.22 g eicosapentaenoic acid (EPA) per day; Probiotics 1x10^10 CFU each of *Lactobacillus rhamnosus* HN001 and *Bifidobacterium animalis ssp. lactis* 420, daily	T1	Placebo	Secondary analysis	Inflammatory markers; Lipid profile; GDM	NR	Fish oil changed serum fatty acids; no clinical effect

57	Soldavini et al. 2022 [106] Italy	RCT - triple blind, placebo controlled	40 17/ 23	Pregnant women diagnosed with gestational diabetes mellitus	combination	omega-3 2.4 g/day; anthocyanins 108 mg/day; alpha-cyclodextrins 15 g/day	T2	Placebo	To assess the effect of nutraceutical supplements (omega-3 fatty acids, anthocyanins, and alpha-cyclodextrins) in GD patients	Inflammatory markers; Lipid profile; Glucose metabolism; Preterm birth; Patients requiring pharmacological inter	Birth weight	No reduction in need for pharmacological therapy; poor compliance;
58	Movaghar et al. 2024 [60] Iran	RCT - triple blind, placebo controlled IRCT20110606006709N20	128 64 / 64	Pregnant women with mild preeclampsia at gestational age 24 weeks or more	combination	>1×10⁹ CFU daily probiotics (lactobacilli, bifidobacterial and streptococci), along with fructo-oligosaccharide pre-biotics	T2/T3	Placebo	Primary: BP and pregnancy duration	Preeclampsia/GH; Preterm birth; Platelet count and serum levels of liver enzymes	NR	Synbiotic lowered systolic and diastolic BP in mild pre-eclampsia<br><br>SBP aMD −13.54 (95% CI −22.07 to −5.01); DBP aMD −10.30 (−15.90 to −4.70)
59	Zahiri et al. 2016 [71] Iran	RCT	540 270/ 270	Healthy pregnant women	zinc	15 mg/day	T2	No treatment	Primary: birth weight	Preeclampsia/GH; Preterm birth; GWG; Spontaneous abortion; PROM;	Birth weight; Macrosomia; Apgar; Head circumference; Birth length;	Zinc 15 mg/day did not improve pregnancy outcomes.
60	Shahnazi et al. 2017 [66] Iran	RCT - double blind, placebo controlled IRCT201311296709N14	92 46 / 46	Healthy pregnant women with a history of PPROM and singleton pregnancy	zinc	40 mg/day	T2	Placebo	Primary: PPROM frequency;	GI symptoms; Preterm birth; Mode of delivery (c-section)	Birth weight; Head circumference	Zinc 40 mg/day did not prevent PPROM/PROM; 3.7% dropout for diarrhoea
61	Roshanravan et al. 2018 [63] Iran	RCT - double blind, placebo controlled IRCT201212265670N6	46 23/23	Pregnant women with impaired glucose tolerance (IGT)	zinc	30 mg/day	T2	Placebo	To evaluate the effect of zinc supplementation on Vaspin and IL-6 levels in pregnant women with impaired glucose tolerance (IGT)	Inflammatory markers; Preterm birth; Vaspin levels	NR	Zinc reduced inflammatory parameters/vaspin in IGT

↳	Roshanravan et al. 2019 [64] Iran	RCT - double blind, placebo controlled IRCT201212265670N6	46 23/23	Pregnant women with impaired glucose tolerance test results	zinc	30 mg/day	T2	Placebo	To evaluate the effects of zinc supplementation on serum leptin, visfatin, tumor necrosis factor-α (TNF-α), adiponectin and zinc-α2-glycoprotein (ZAG) levels in pregnant women with impaired glucose tolerance test (IGTT) results.	Inflammatory markers; GI symptoms; Preterm birth; Leptin levels	Birth weight; neonatal height, head circumference	Zinc: adipokines and neonatal anthropometry; no side effects reported
62	Mesdaghinia et al. 2021 [56] Iran	RCT - double blind, placebo controlled IRCT20170513033941N33	52 26 /26	Pregnant women primigravida at risk for intrauterine growth restriction	zinc	30 mg/day zinc elemental (zinc gluconate 233 mg)	T2	Placebo	Primary: pulsatility index and TAC.	Inflammatory markers; Lipid profile; Glucose metabolism; Total antioxidant capacity; Plasma malonylaldehyde; Pulsatility index	NR	Zinc reduced hs-CRP, MDA, insulin, HOMA-IR; no effect on pulsatility index.<br><br>hs-CRP β −1.17 mg/L (95% CI −1.77 to −0.57); insulin β −1.33 (−2.00 to −0.67)
63	Gao et al. 2026 [75] China	Retrospective cohort	205 104/101	Pregnant women with chronic hypertension	zinc	20 mg/day of elemental zinc	T1	No treatment	Primary endpoint: pre-eclampsia incidence	Inflammatory markers; Preeclampsia/GH; Preterm birth; Mode of delivery (c-section);	Birth weight; Apgar;	First-trimester zinc associated with lower pre-eclampsia in chronic hypertensive pregnancies.<br><br>PE 21.15% vs 38.61%, RR 0.77 (95% CI 0.63–0.95), P=0.01, as reported by the authors (the RR is not arithmetically consistent with the percentages, which give ≈0.55)
64	Bullarbo et al. 2018 [92] Sweden	RCT - double blind, placebo controlled ISRCTN13890849	176 83/93	Nulliparous healthy pregnant women	magnesium citrate	400 mg/day	T1	Placebo	Primary: increase of diastolic BP ≥15 mmHg	Inflammatory markers; GI symptoms; Preeclampsia/GH; Mode of delivery (c-section); Systolic and diastolic blood pressure	Birth weight; Apgar; pH in the arterial umbilical cord; Birth length	Mg 400 mg/day did not prevent diastolic BP rise; no effect on IL-6/CRP; nausea main dropout reason
65	Araújo et al. 2020 [110] Brazil	RCT - double blind, placebo controlled NCT02032186	829 407/422	Pregnant women with singleton pregnancies at higher risk for preterm birth or adverse perinatal outcomes	magnesium	300 mg/day (magnesium citrate)	T1	Placebo	Primary: composite perinatal outcome	Preeclampsia/GH; Preterm birth	SGA; NICU admission	BRAMAG: magnesium citrate did not reduce perinatal or maternal composite outcomes<br><br>Perinatal composite aOR 1.10 (95% CI 0.72–1.68); maternal composite aOR 1.29 (0.83–2.00)
↳	Araújo et al. 2020 [112] Brazil	Secondary analysis of RCT NCT02032186	318 159/159	Low-income pregnant women	magnesium	300 mg/day (magnesium citrate)	T1	Placebo	Primary: pre-eclampsia	GI symptoms; Preeclampsia/GH; Hepatic enzymes; Renal impairment; Pulmonary edema.	NR	Secondary analysis (low-income subset): magnesium did not reduce pre-eclampsia (OR 0.90 (95% CI 0.48-1.69, p=0.747). Minor, nonsignificant GI side effects.

66	Aslfalah et al. 2019 [36] Iran	RCT - double blind, placebo controlled IRCT20160111025949N6	60 30 / 30	Pregnant women with gestational diabetes mellitus	alpha lipoic acid	100 mg/day	T2	Placebo	To explore possible beneficial effects of ALA supplementation in women with GDM	Glucose metabolism; Preterm birth; GWG; Hepatic transaminase, gamma-GT, alkaline phosphatase	NR	Fasting blood sugar (P < 0.001), gamma-glutamyltransferase (P < 0.001) and alanine transaminase (P = 0.031) were decreased in the drug group after the intervention.
↳	Aslfalah et al. 2019 [37] Iran	RCT - double blind, placebo controlled IRCT20170912 25949N5	60 30 / 30	Pregnant women with gestational diabetes mellitus	alpha lipoic acid	100 mg/day	T2	Placebo	To evaluate the effects of ALA supplementation on maternal circulating values of adiponectin, leptin and their ratios in women with GDM	Glucose metabolism; GWG; Adiponectin , adiponectin/leptin ratio, lleptin/adiponectin ratio, adiponectin/HOMA-IR ratio; malondialdehyde/total antioxidant capacity	NR	FBS, (p < .001), HOMA-IR (p < 0.001), MDA/TAC (p < .001), and L/A (p = .008) were decreased while values of adiponectin (p = 0.011), A/L (p = 0.001), and A/H (p < 0.001) were increased in the drug group after the intervention.
↳	Aslfalah et al. 2020 [38] Iran	RCT - double blind, placebo controlled IRCT20170112925949N4	60 30 / 30	Pregnant women with gestational diabetes mellitus	alpha lipoic acid	100 mg/day	T2	Placebo	To investigate the effects of alpha-lipoic acid (ALA) on the maternal circulating values of lipid profile and lipid ratios in women with GDM.	Lipid profile; Glucose metabolism; Preeclampsia/GH; GWG; Thiobarbituric acid-reactive substances	NR	TyG index (p < 0.001), TG (p = 0.006), TG/HDL-C (p = 0.003), and AIP (p = 0.005) decreased significantly in the ALA group after the intervention.
67	Mandani et al. 2021 [55] Iran	RCT - double blind, placebo controlled IRCT20181113041639N1	60 30 / 30	Pregnant women newly diagnosed with gestational diabetes mellitus	alpha lipoic acid	300 mg/day	T2	Placebo	To investigate the effects of ALA supplementation on some oxidative stress biomarkers in women with GDM	Glucose metabolism; GWG; Total antioxidant capacity; Catalase; Glutathione;	Birth weight; Birth length; Head circumference;	ALA 300 mg/day improved FBS and oxidative markers(FBS (p =0.001), TAC (p < 0.001), OSI (p =0 .003), TAG (p =0 .001) and catalase (p < 0.001) were improved significantly in the drug group. No difference in birth weight, length, head circumference
68	Fadinie et al. 2019 [74] Indonesia	RCT - double blind, placebo controlled	47 23 / 24	Pregnant women diagnosed with preeclampsia scheduled for caesarean section	curcumin	100 mg/day	T3	Placebo	To evaluate the effect of perioperative curcumin administration on IL-10 and COX-2 in preeclamptic patients undergoing caesarean section under spinal anaesthesia.	Inflammatory markers; Preeclampsia/GH; Mode of delivery(c-section)	NR	Single 100 mg curcumin before caesarean under spinal anaesthesia: no significant difference in serum COX-2 or IL-10;
69	Bahramian et al. 2018 [40] Iran	RCT - triple blind, placebo controlled IRCT20160404327N36	75 37/ 38	Pregnant women with gestational diabetes mellitus, singleton pregnancy	ginger	500 mg ginger rhizome per capsule, twice daily	T2	Placebo	To assess the effect of ginger capsules on the control of blood sugar in gestational diabetes.	Glucose metabolism; GI symptoms; Vaginal hemorrhage; Backache; Skin rash; Dizziness;	NR	Ginger reduced 2-h postprandial glucose and insulin dose (P < 0.05); FBS not different; mild GI AEs

70	Laekeman et al. 2021 [100] Belgium	Uncontrolled prospective single-arm feasibility study	51(enrolled)/ 44 exposed	Pregnant women with gastrointestinal discomfort	ginger	100 mg/day maximum (2 tablets × 50 mg each)	T1	Not applicable	Primary objective: maternal complications and neonatal malformations	GDM; Preeclampsia/GH; Preterm birth; Spontaneous abortions; Hyperemesis gravidarum; Cholestasis; Proteinuria	Congenital anomalies;	51 enrolled, 44 exposed (1–55 tablets): 2 spontaneous abortions (1 unexposed), 10 premature births, 4 hip immaturity, 2 minor malformations; no relation to number of tablets
71	Zhang et al. 2017 [89] China	RCT - double blind, placebo controlled	326 176/ 150	Pregnant women with gestational diabetes mellitus	EGCG	500 mg/day	T3	Placebo	To investigate the effect of daily dietary EGCG supplementation on both the maternal and neonatal treatment outcomes in GDM	Glucose metabolism; GWG	Birth weight; Apgar; Hypoglycaemia; Macrosomia; Respiratory distress	EGCG 500 mg/day (≥95% purity) improved maternal glycaemic control and neonatal outcomes versus placebo (hypoglycaemia P =0.01, LBW P=0.03). Apgar scores were significantly higher in the EGCG group compared to the placebo group [1-min Apgar: 9.7 (0.4) in EGCG, 9.4 (0.3) in placebo, P = 0.04; 5-min Apgar: 10.7 (0.6) in EGCG, 9.3 (0.5) in placebo, P = 0.02]
72	Shi et al. 2018 [81] China	RCT - double blind, placebo controlled	304 148 / 156	Pregnant women with severe pre-eclampsia	EGCG	100 mg/dose, up to 5 doses	T3	Placebo	Primary: time to BP control; interval before new crisis	GI symptoms; Preeclampsia/GH; Adverse effects (tachycardia, headache, dizziness, chest pain, hypotension, shortness of breath)	Birth weight; Apgar;	EGCG (≥98% purity) shortened time to BP control and prolonged interval to next crisis as adjunct to nifedipine (Time to control blood pressure significantly shorter in NIF+EGCG (31.2±16.7 minutes) vs NIF+placebo (45.3±21.9 minutes), P=0.03, 95% CI 9.7-18.5 minutes. Interval time before new hypertensive crisis significantly prolonged in NIF+EGCG (7.2±2.9 hours) vs NIF+placebo (4.1±3.7 hours), P=0.02, 95% CI 2.3-3.9 hours.); maternal AEs and neonatal outcomes similar
73	Ding et al. 2017 [73] China	RCT - double blind, placebo controlled	349 174/ 175	Pregnant women with singleton pregnancy and severe preeclampsia	resveratrol	50 mg/dose, up to 5 doses per session (up to 250 mg total per episode), adjuvant to nifedipine	T3	Placebo	Primary: time to BP control and time before new hypertensive crisis; secondary: doses needed, maternal and neonatal adverse effects	GI symptoms; Preeclampsia/GH; Adverse effects (tachycardia, headache, dizziness, chest pain, hypotension, shortness of breath)	Birth weight; Apgar	Resveratrol adjunct to nifedipine shortened time to BP control and delayed next crisis (NIFE+RESV significantly reduced time to control BP (35.6±18.7 min vs 51.1±22.4 min, P=0.01); time before new hypertensive crisis significantly longer in NIFE+RESV (8.0±2.1 h vs 5.5±1.8 h, P=0.02)); no difference in maternal or neonatal adverse effects;

74	Malvasi et al. 2017 [101] Italy	RCT - double blind, placebo controlled	104 35 /35 / 35	Overweight pregnant women	resveratrol	Group 1: 80 mg trans-resveratrol (Revifast®), adjuvant to 200 mg myo-inositol, 500 mg D-chiro-inositol per day; Group 2: 138 mg myo-inositol, 550 mg D-chiro-inositol per day, Group 3: placebo	T2	Active comparator, Placebo	To evaluate the metabolic effects of resveratrol in second trimester of pregnancy	Lipid profile; Glucose metabolism; GDM; No differences in systolic and diastolic pressure	NR	The supplementation of Trans-resveratrol, in addition to DCI/MI in overweight pregnant woman with an elevated fasting glucose, lowered glucose, TC, LDL, TG vs placebo
75	Uchiyama-Tanaka et al. 2024 [83] Japan	RCT - open label	20 10 / 10	Pregnant women with low-grade inflammation	chlorella	6000 mg/day	T2	No treatment	To evaluate the efficacy and safety of orally administered Chlorella supplementation in pregnant women with low-grade inflammation and assess its effects on constipation, inflammation markers, and fetal outcomes.	Inflammatory markers; GI symptoms; Preeclampsia/GH; Hemoglobin and ferritin levels; Iron levels	Birth weight; Apgar; Birth length	Chlorella 6 g/day reduced constipation; 2 control withdrawals for severe PIH/eclampsia; neonatal outcomes not different. The supplement was well-tolerated and safe, with no adverse effects reported.
76	Sen et al. 2024 [117]<br><br>United States	Double-blind, placebo-controlled randomized controlled trial<br><br>NCT02802566	128<br><br>52 / 46	Pregnant women with obesity (BMI >=30 kg/m2) enrolled <13 weeks	combination	Vitamin C 90 mg/day (ascorbic acid); vitamin E 30 alpha-TU/day (d-alpha-tocopherol acetate)	T1	Placebo	Primary: Micronutrient concentrations and markers of inflammation and oxidative stress markers	Inflammatory markers; Oxidative stress; GDM; PH; GWG; Mode of delivery (c-section); Post-partum infection and thrombosis; GI symptoms (AEs)	Preterm birth; Birth weight; Weight, length and BMI z-scores; Apgar; SGA	Vitamin B6 concentrations increased; vitamins C, E and folate unchanged; no effect on inflammation, oxidative stress or maternal or neonatal clinical outcomes; AEs minor GI, balanced<br><br>Hypertension in pregnancy OR 2.7 (0.9-8.4); GDM OR 1.0 (0.3-2.8); caesarean OR 1.3 (0.6-2.9); preterm OR 1.4 (0.5-4.3); birth weight difference 0.0 kg (-0.3 to 0.3)

						; vitamin B6 18 mg/day (pyridoxine HCl); folic acid 800 µg/day						
77	Zhang et al. 2016 [88]<br><br>China	Randomized, double-blind, placebo-controlled	133<br><br>20 / 38 / 38 / 37	Pregnant women with GDM, pre-pregnancy BMI <=30 kg/m2, not on insulin, no vitamin D supplementation in previous 6 months	vitamin D	200 IU/day; 50,000 IU/month (2,000 IU/day for 25 days); 50,000 IU every 2 weeks (4,000 IU/day for 12.5 days)	T2	Placebo	Primary outcome: difference in serum 25(OH)D;glucose metabolism, lipids, inflammation and oxidative stress	FPG; insulin; HOMA-IR; total cholesterol change; triglycerides; hs-CRP; TAC; GSH; weight and BMI; 25(OH)D; calcium;<br><br>adverse effects of vitamin D (headache, vomiting) monitored monthly	NR	High and medium doses reduced insulin and HOMA-IR and total cholesterol change vs placebo(P<0.01). FPG and triglycerides unchanged; hs-CRP not reduced; TAC and GSH increased with all doses (dose-dependent)<br><br>AEs monitored (headache, vomiting at monthly visits) but results not reported
78	Fatima et al. 2019 [128]<br><br>Pakistan	Controlled clinical trial	60<br><br>30 / 30	Pregnant women with GDM requiring insulin, 32nd week of gestation	zinc	Zinc sulfate 25 mg/day (oral)	T3	Placebo	Comparisons: insulin dose requirement, macrosomia and pre-eclampsia	Insulin dose and change in insulin dose; pre-eclampsia; Mode of delivery (c-section);	Birth weight; macrosomia (large neonates)	Insulin dose increase during follow-up lower with zinc (Insulin dose change 8.76 +/- 9.63 vs 17.53 +/- 10.54 IU (P=0.001)); macrosomia lower with zinc (macrosomia 2/30 (6.7%) vs 10/30 (33.3%), P=0.01) no difference in pre-eclampsia or caesarean section<br><br>AEs not reported
79	Nausheen et al. 2021 [129]<br><br>Pakistan	RCT - double blind<br><br>NCT02215213	253<br><br>79 / 85 / 89	Pregnant women <16 weeks of gestation, singleton	vitamin D	Vitamin D3 syrup 4000 IU/day or 2000 IU/day vs 400 IU/day	T1	Active comparator: vitamin D3 400 IU/day	Primary: vitamin D and calcium deficiency; pregnancy and birth outcomes (pre-eclampsia, GDM, preterm birth, low birth weight, stillbirth)	Serum 25(OH)D, calcium, phosphorus, alkaline phosphatase; pre-eclampsia; GDM; hypercalcaemia; hypervitaminosis D; adverse events and serious adverse events;	Preterm birth; low birth weight; stillbirth; early neonatal death; neonatal 25(OH)D, calcium; neonatal abnormalities	Vitamin D 4000 IU/day reduced maternal and neonatal vitamin D deficiency vs 400 IU/day (vitamin D deficiency at endline 75.9% vs 84.9% vs 90.2% (P=0.006)); pregnancy and birth outcomes did not differ between groups;<br><br>No adverse events, hypercalcaemia or hypervitaminosis D
80	Sunarno et al. 2023 [82]<br><br>Indonesia	RCT - open label<br><br>ISRCTN87262826	108<br><br>36 / 36 / 36	Pregnant women at 20 weeks of gestation, normotensive, serum	vitamin D	Vitamin D3 1000 IU/day for 17 weeks	T2	No treatment	Compare effects on calcidiol levels, blood pressure and newborn anthropometric indices	Serum calcidiol; systolic and diastolic blood pressure; GWG	Birth weight; birth length; head circumference; low birth weight	Vitamin D increased calcidiol and maintained/lowered systolic blood pressure (systolic BP 105.8 -> 104.6 mmHg within vitamin D group, P=0.019;), birth weight highest in the vitamin D group, birth length increased (Birth weight 3172 +/- 182 (vitamin D) vs 3091

				calcidiol <30 ng/mL								+/- 363 (sun) vs 2861 +/- 208 g (control), P<0.001; adjusted effect of vitamin D on birth weight +302.21 g (SE 70.64), P<0.001; on birth length +1.10 cm (SE 0.42), P=0.011; on calcidiol +12.23 ng/mL (SE 0.86), P<0.001)<br><br>AEs: not reported

Notes: ↳ companion publication from the same trial population; NR = not reported; GDM = gestational diabetes mellitus; GH = gestational hypertension; SBP = systolic blood pressure; GWG = gestational weight gain; GI = gastrointestinal; TAC = total antioxidant capacity; SGA = small for gestational age; LGA = large for gestational age; EGCG = epigallocatechin-3-gallate; SAE = serious adverse event; AE = adverse event. T1/T2/T3 = first/second/third trimester; Trimester = trimester in which supplementation was initiated, as reported; labels spanning trimesters denote an initiation window that crossed a trimester boundary or was not specified. Follow-up: outcomes are reported to delivery or the neonatal period unless stated; outcomes of parent trials not reported in the included publication are noted but not counted.
